# Structural basis for full-spectrum inhibition of translational functions on a tRNA synthetase

**DOI:** 10.1038/ncomms7402

**Published:** 2015-03-31

**Authors:** Pengfei Fang, Xue Yu, Seung Jae Jeong, Adam Mirando, Kaige Chen, Xin Chen, Sunghoon Kim, Christopher S. Francklyn, Min Guo

**Affiliations:** 1 Department of Cancer Biology, Scripps Research Institute, Scripps Florida, 130 Scripps Way, Jupiter, Florida 33458, USA; 2 Medicinal Bioconvergence Research Center, Seoul National University, Seoul 151-742, Korea; 3 Department of Biochemistry, University of Vermont College of Medicine, 89 Beaumont Avenue, Burlington, Vermont 05405, USA

## Abstract

The polyketide natural product borrelidin displays antibacterial, antifungal, antimalarial, anticancer, insecticidal and herbicidal activities through the selective inhibition of threonyl-tRNA synthetase (ThrRS). How borrelidin simultaneously attenuates bacterial growth and suppresses a variety of infections in plants and animals is not known. Here we show, using X-ray crystal structures and functional analyses, that a single molecule of borrelidin simultaneously occupies four distinct subsites within the catalytic domain of bacterial and human ThrRSs. These include the three substrate-binding sites for amino acid, ATP and tRNA associated with aminoacylation, and a fourth ‘orthogonal’ subsite created as a consequence of binding. Thus, borrelidin competes with all three aminoacylation substrates, providing a potent and redundant mechanism to inhibit ThrRS during protein synthesis. These results highlight a surprising natural design to achieve the quadrivalent inhibition of translation through a highly conserved family of enzymes.

## Abstract (web summary)

Borrelidin is an antibiotic with antimicrobial, antifungal, antimalarial and immunosuppressive activity that targets threonyl-tRNA synthetase. Here the authors show that borrelidin functions by preventing binding of all three ThrRS substrates and inducing a distinct, non-productive, conformation of the enzyme.

## Graphical abstract

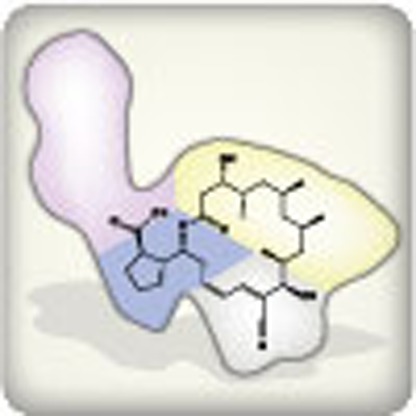

Borrelidin (BN) was originally isolated from culture broths of the marine antagonistic actinomycete strain *Streptomyces rochei* or *Streptomyces parvulus*, and exhibits multiple biological functions on a wide range of organisms^1^,^2^,^3^. BN activities include antibacterial, antifungal, antimalarial, insecticidal and herbicidal, and recently anti-vascular endothelial growth factor-induced angiogenesis activities^4^,^5^,^6^,^7^,^8^. BN protects mice from lethal malaria infection^9^. The antimalarial activities of BN against drug-sensitive FCR3 strain and drug-resistant K1 strain of *P. falciparum* (IC_50_: 1.8–1.9 nM) are even more effective than the most potent antimalarials currently used in clinics, including artemether, artesunate and chloroquine^10^. BN inhibits *in vivo* blood vessel formation and angiogenesis of the rat aortic tube with an IC_50_ of 0.8 nM (ref. 11). BN also inhibited spontaneous lung metastasis of B16-BL6 melanoma at the same dose that inhibited angiogenesis^12^. These diverse activities stand in marked contrast to the apparent unitary nature of its target, the essential translation enzyme threonyl-tRNA synthetase (ThrRS or TARS)^13^,^14^,^15^,^16^.

ThrRS is one of the 20 (in general) aminoacyl-tRNA synthetases (AARSs) that are essential enzymes responsible for charging corresponding amino acids to their cognate tRNAs and providing the correct substrates for high-fidelity protein synthesis^17^,^18^. A two-step aminoacylation reaction involving the binding of amino acid and activation of ATP, followed by a transfer of the aminoacyl-group from the high-energy intermediate aminoacyl-adenylate (AA-AMP) to the 3′-OH of tRNA, is essential for establishing the genetic-code linkage, and is a fundamental process in all cellular life^18^. As the first characterized AARS inhibitor, BN was initially linked to AARS through its antibiotic action in microorganisms, which involves selective inhibition of threonine incorporation in tRNA^4^.

The inhibition of BN was soon confirmed in a variety of ThrRSs from bacteria to human cells^4^,^5^,^19^. BN suppresses threonyl-tRNA formation in *Escherichia coli*^4^, yeast^5^ and human acute lymphoblastic leukaemia cells^19^. The subsequent rise in the levels of uncharged tRNA in acute lymphoblastic leukaemia cells further leads to the induction of the general control nonderepressible-2 kinase stress responsive pathway, and eventual cellular apoptosis^19^. Other reports have demonstrated that an expression level increase or sequence alteration of ThrRS is associated with BN resistance in *E. coli*, yeast and Chinese hamster ovary cells (CHO cells)^14^,^15^,^16^. BN-resistant *E. coli* K12 strains can be selected with a frequency of 1 × 10^−8^, and include strains with an increased level of wild-type ThrRS, and strains that harbour a mutated *thrS* gene^14^. BN-resistant CHO cells also displayed threefold increased level of ThrRS activity^16^. In fact, BN inhibits the threonine activation step of bacterial ThrRS with a *K*_i_ value of about 4 nM (ref. 20), and a *K*_i_ of about 7 nM for human ThrRS (Supplementary Fig. 1a), placing it among the most potent AARS inhibitors described (Table 1). How BN acts on ThrRS to potently control its function across a variety of species remains unclear.

Here we present a detailed structural and functional analysis of the binding of BN to ThrRS. We determined the crystal structures of both human and *E. coli* ThrRS in complex with BN, and identified a unique structural inhibition mechanism of BN against ThrRS. BN occupies a significant fraction of the total volume of the ThrRS enzymatic pocket, physically excluding all three of the physiological substrates of ThrRS, namely _L-_threonine (_L-_Thr), ATP and tRNA. Inhibition of translation by BN can be rescued by the addition of each substrate, indicating that BN acts as a triple-competitive inhibitor. Although occupying the canonical active site cavity, BN also extends into a fourth ‘orthogonal’ pocket. This fourth pocket is not evident in the substrate-bound ThrRS structures, underscoring the induced-fit nature of BN’s interaction with ThrRS. Occupancy of the fourth subsite further intervenes the aminoacylation activity of ThrRS, producing a redundant mechanism for inhibition of protein translation. These results highlight the surprising design of a natural polyketide to achieve quadrivalent binding and inhibition of a tRNA synthetase in two of the three kingdoms of life.

## Results

### Structural basis of specific ThrRS–BN recognition

**Figure 1:**
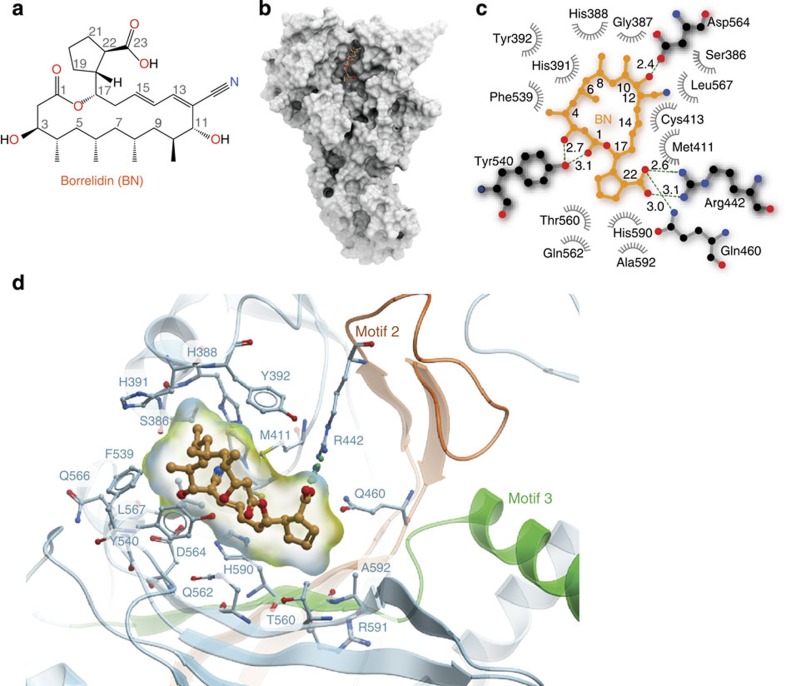
Structural basis of specific ThrRS–Borrelidin (BN) recognition. (**a**) Chemical structure of BN. (**b**) BN is deeply buried in an induced-fit pocket of human threonyl-tRNA synthetase (ThrRS). The compound is shown as orange sticks; the surface view of one human ThrRS monomer is shown in grey. (**c**) Two-dimensional presentation of BN binding in human ThrRS. BN and hydrogen-bonding residues are shown in stick representations, and other residues within 4.5 Å of BN are shown in grey. (**d**) Zoom-in view of BN localization in the conserved catalytic core of ThrRS. The classical motifs 2 and 3 in class II aminoacyl-tRNA synthetases (AARSs) are coloured in orange and green, respectively. BN and interacting residues are shown as sticks.

To elucidate the species-independent, full-spectrum inhibitory mechanism of BN, we co-crystallized a fragment of human ThrRS containing the essential catalytic domain and anticodon-binding domain (residues 322–723, Supplementary Fig. 1b) with BN and determined the structure to a resolution of 2.6 Å (Table 2). As a typical class II AARS^21^, human ThrRS forms a dimer through the catalytic domain, with each subunit binding one molecule of BN (Supplementary Fig. 2). The polyketide BN binds tightly in the aminoacylation active site cavity of human ThrRS (Fig. 1a,b), constituted by class II AARS signature motif 2 (residue 432–469), motif 3 (585–613)^22^ and surrounding hydrophobic loops 386–393, 411–413, 538–540 and 564–567 (Fig. 1c,d). The plane of the 18-membered ring in BN fits perpendicularly to the seven-stranded β-sheet of the catalytic domain (Fig. 1d).

We also crystallized an *E. coli* ThrRS fragment containing the catalytic and anticodon-binding domains with BN (Supplementary Fig. 3a). The structures of human ThrRS and *E. coli* ThrRS exhibit high similarity, with an r.m.s.d. (root mean square distance) of 0.664 Å for 279 Cα atoms superimposed over the catalytic domains (Supplementary Fig. 3b). Consistent with the 48% sequence identity between human and *E. coli* ThrRS’s in the catalytic domain, BN is bound in the active site cavity with a nearly identical structure in human and *E. coli* ThrRS–BN complex structures. The binding modes of BN in both the human and *E. coli* ThrRSs are therefore closely similar. The r.m.s.d. of all 35 non-hydrogen atoms of the bound BNs from the two structures is only 0.314 Å. By virtue of its location deep within the catalytic pocket, BN interacts with the enzyme in all directions (Fig. 1c and Supplementary Fig. 4). Thirty-three of its thirty-five non-hydrogen atoms (except C4 and C8) form six hydrogen bonds and a total of 155 interactions with the protein within 4.5 Å (Fig. 1d). Eighteen residues from the ThrRS active site contribute to these interactions, fifteen of which are strictly conserved in bacterial, fungal, plasmodium and other eukaryotic ThrRSs (Supplementary Figs 5 and 6). Therefore, the inhibition mechanism of ThrRS by BN appears to be conserved from bacteria to human.

In contrast, archaeal ThrRSs are resistant to BN^20^. Out of the 18 key BN-interacting residues in bacteria and eukaryotes, 12 are altered in archaeal ThrRS, and may account for its kingdom specificity (Supplementary Figs 5,7 and 8). These residues include S386, G387, H388, H391, Y392, M411, Q460, F539, Y540, Q566, L567 and A592 (numbering by human ThrRS). For instance, Q460 on the floor of motif 2 in human ThrRS forms a key hydrogen bond with the only carboxylic group of BN. The corresponding residue is a methionine in archaeal ThrRS, representing a critical hydrogen bond with BN would be lost (Supplementary Figs 5 and 8). Similarly, A592 in motif 3 in human ThrRS forms a hydrophobic interaction with the C20 methylene groups of BN that would not be possible in the archaeal enzymes, owing to a substitution by hydrophilic serine (Supplementary Figs 5 and 8). Among other hydrophobic interactions, van der Waals contacts between Q566, L567, S386, G387 and H388 on the back wall of the cavity with the 8′ methyl and 12′ cyano groups of BN (Fig. 1c and Supplementary Fig. 4) would be prevented with archaeal ThrRS, because of non-conserved substitutions of these residues (Supplementary Figs 4 and 7a).

The incompatibility of BN with archaeal ThrRSs may be associated with the early separation of archaeal ThrRS from bacterial and eukaryotic ThrRS in evolution^23^. Similarly, archaeal ThrRSs also contain a unique D-amino-acid deacylase-like editing domain that is orthogonal to the typical bacterial/eukaryotic-editing domain^24^. In support of the discriminative role of these residues, mutations at H309 and L489 in *E. coli* ThrRS (corresponding to H388 and L567 in human) confer 300- to 1,000-fold increases in *K*_i_, representing significant resistance to BN^20^ (Supplementary Fig. 7c,d). In summary, the preponderance of amino-acid substitutions at key BN-interacting residues in archaeal ThrRS provides a highly redundant basis for the essentially kingdom-wide resistance to BN (Supplementary Fig. 8).

### BN simultaneously occludes the 3 substrate-binding sites

**Figure 2:**
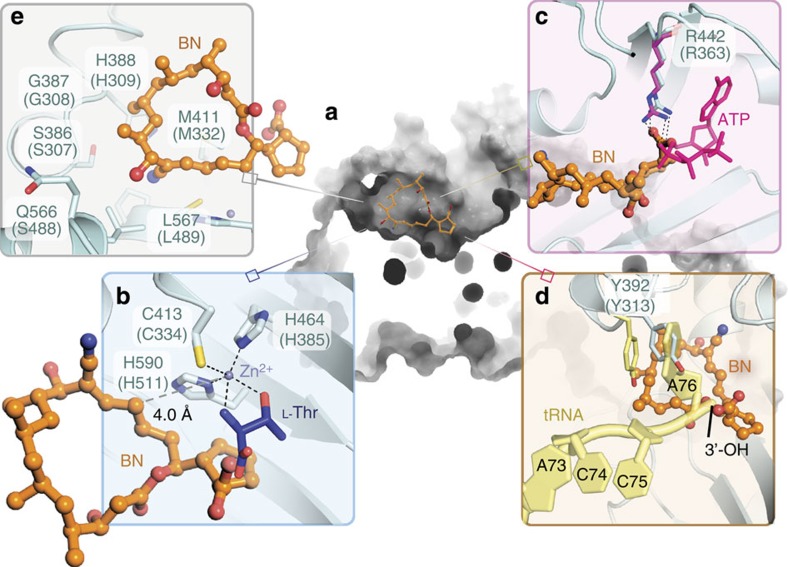
BN excludes all substrates of ThrRS for protein translation. (**a**) Section view of BN (orange) occupying the catalytic site cavity of ThrRS. (**b**) Close-up view of _L-_Thr pocket of superimposed *E. coli* ThrRS–_L-_Thr structure (blue, PDB: 1EVK) with ThrRS–BN structure (cyan). Numbering of residues is according to human ThrRS and the corresponding *E. coli* ThrRS residues are shown in parenthesis. (**c**) Close-up view of ATP pocket of superimposed *Staphylococcus aureus* ThrRS–ATP structure (magenta, PDB: 1NYR) with ThrRS–BN structure (cyan). (**d**) Close-up view of tRNA pocket of superimposed *E. coli* ThrRS–tRNA^Thr^ structure (yellow, PDB: 1QF6) with ThrRS–BN structure (cyan). (**e**) Close-up view of extra hydrophobic pocket occupied by BN (orange), which is beyond the substrate-binding pockets.

The two-step reaction of aminoacylation involves the binding of amino acid and activation of ATP, followed by a transfer of the activated amino acid to the 3′-OH of appropriate tRNA^25^. However, the polyketide structure of BN does not resemble any of the substrates of ThrRS. To understand the mechanism of inhibition of BN on ThrRS aminoacylation activity, we compared the ThrRS–BN structure with ThrRS structures in which one or more substrates are bound. With respect to _L_-Thr, BN’s location in the enzymatic centre of each ThrRS subunit (Fig. 2) creates a steric clash between the BN cyclopentanecarboxylic group (C18–23) and the amino-acid carboxyl group (Fig. 2b). Ten of eighteen BN-interacting residues are involved in the interactions with _L_-Thr during normal aminoacylation (Supplementary Figs 5 and 9), including two residues H590 and C413 that chelate the conserved zinc atom^26^ (Fig. 2b). In ThrRS complexes with bound _L-_Thr, these latter residues collaborate with H464 (which does not interact with BN) to chelate the essential zinc atom that recognizes the β-OH group of _L_-Thr^27^. BN also triggers side chain rearrangements that disrupts the _L-_Thr-binding site, stabilizing rotamer conformations of T560, Y540 and Q562 that would be incompatible with _L-_Thr binding (Supplementary Fig. 9). Co-crystallization of the ThrRS–BN complex in the presence of excess _L_-Thr (5 mM), ATP analogue AMP_C_PP (5 mM), and Mg^2+^ (10 mM) revealed density only for BN (2 mM) but not for _L_-Thr or AMP_C_PP. This suggests that _L_-Thr and BN cannot bind to ThrRS simultaneously (Supplementary Fig. 3c,d).

Further structural inspection reveals that BN also simultaneously occludes binding of ATP and tRNA. The C22–23 atoms of the cyclopentanecarboxylic group of BN overlap with the superimposed α-phosphate group of ATP (Fig. 2c). Notably, the carboxyl acid group on BN precludes a key interaction in aminoacylation by forming a bifurcated salt-bridge with R442 (Fig. 2c). As a characteristic residue conserved in all class II AARSs, R442 activates AA-AMP formation by stabilizing the pentacoordinate transition state of the α-phosphate^28^,^29^. By interacting with the guanidine group of R442, BN prevents R442 binding to _L_-Thr and ATP, thereby blocking the formation of the threonyl-adenylate (Thr-AMP; Fig. 2c). C4–5 of BN further pushes Y392 into the tRNA^Thr^ A76 adenosine-binding pocket, whereas in the tRNA-bound structure of the *E. coli* ThrRS, the corresponding Y313 stacks with the adenine ring of A76 for aminoacylation^27^ (Fig. 2d). In addition, the C1–3 portion of BN also creates a steric clash with the A76 ribose of tRNA^Thr^ (Fig. 2d), thereby preventing the productive binding of tRNA. All together, these structural observations indicate that BN occupies the central ThrRS active site cavity that joins the three substrate-binding pockets for _L-_Thr, ATP and tRNA, and effectively excludes substrate binding to ThrRS (Fig. 2).

### BN competes with all 3 substrates of ThrRS for translation

**Figure 3:**
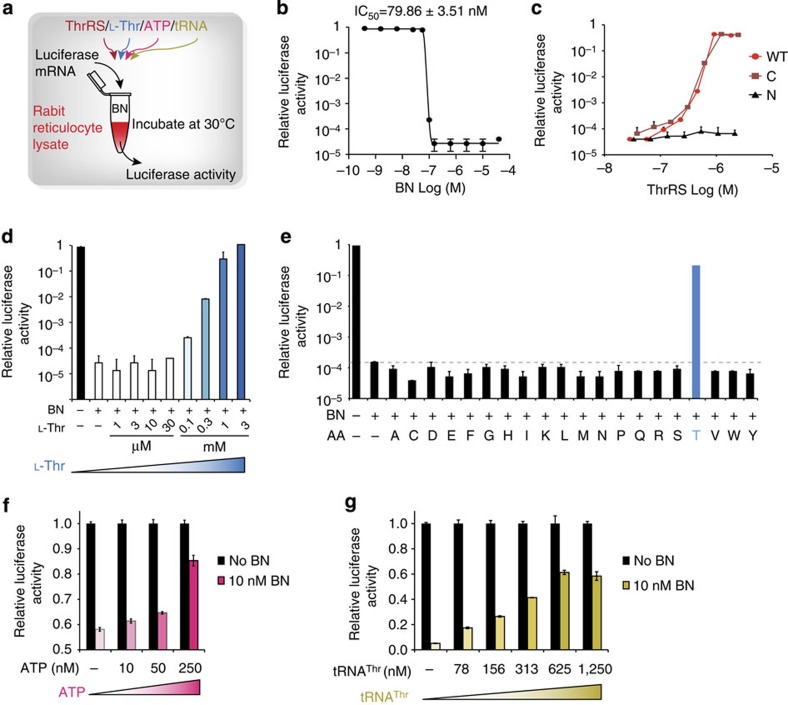
BN competes with all three substrates of ThrRS for protein translation. (**a**) Rabbit reticulocyte lysate (RRL) was incubated with luciferase mRNA and translation of luciferase enzyme was quantified in a luminescence assay. BN was used to inhibit the translation, whereas human ThrRS protein or ThrRS substrates were used to rescue the inhibition by BN. (**b**) Serial diluted BN (0.08 nM–100 μM) was added to inhibit the translation of luciferase mRNA. (**c**) Human ThrRS full-length (WT) and fragments (*N*: 1–322; C: 322–723) were added to rescue the protein translation inhibited by 250 nM BN. (**d**) Serial diluted _L_-Thr (1 μM–3 mM) was added to rescue the protein translation inhibited by 250 nM BN. (**e**) Similarly, individual amino acids were added to the final concentration of 1 mM in the reaction to rescue the inhibited translation by 250 nM BN. (**f**,**g**) ATP and tRNA rescued the inhibition of translation by BN in a diluted *in vitro* translation system (see Methods). (**f**) 10–250 nM ATP was tested with or without 10 nM BN. (**g**) *E. coli* expressed human tRNA^Thr^ (78–1250, nM) was assayed with or without 10 nM BN. Luciferase activity was normalized to the corresponding reactions without 10 nM BN. All error bars represent standard deviations (s.d.) of experimental triplicates.

To further explore the mechanism of BN inhibition on protein translation, we used a rabbit reticulocyte lysate-based *in vitro* translation system (Fig. 3a). This system contains all the machinery necessary to translate proteins but lacks endogenous DNA or mRNA, effectively minimizing background translation in the absence of exogenous transcript^30^. BN inhibited the translation of luciferase mRNA in a dose-dependent manner (IC_50_=79.86±3.51 nM, Fig. 3b). The inhibition was rescued by addition of exogenous human full-length ThrRS or C-ThrRS (322–723) alone (Fig. 3c). In contrast, an N-ThrRS construct (1–322) lacking the catalytic domain failed to rescue the translation at any tested concentration, consistent with the absence of BN-binding determinants in this region of the protein (Fig. 3c). Together, these results indicate that BN can inhibit protein translation through the direct binding and inhibition of ThrRS.

To further explore the competitive nature of BN inhibition on ThrRS, we tested the ability of _L-_Thr to compete out the inhibitory effect of BN (Fig. 3d). Translation was recovered in BN-inhibited reactions (250 nM) by the addition of 1 mM _L_-Thr, consistent with the three orders of magnitude weaker binding affinity of _L-_Thr relative to BN. Moreover, only _L_-Thr was able to rescue translation among all 20 amino acids (Fig. 3e). To examine the effect of ATP and tRNA^Thr^, we prepared a diluted system where the reaction sensitivity was increased because of the reduced concentration of the endogenous ThrRS. Again, ATP and tRNA^Thr^ showed a concentration-dependent rescue in the diluted *in vitro* translation system (Fig. 3f,g). In an earlier study, increase of _L_-Thr concentration was observed to suppress BN-induced apoptosis in endothelial cells^31^, whereas limiting _L_-Thr promoted BN sensitivity in cultured CHO cells^16^. Together, these results indicate that the cellular effects of BN are the result of the direct inhibition of ThrRS. By occupying the core site of catalytic cavity, BN competes with all three substrates of ThrRS for translation (Supplementary Movie 1).

### BN occupies the fourth site by its macrolide ring structure

Interestingly, previous studies investigating the BN mechanism of inhibition concluded that it was a noncompetitive inhibitor for _L-_Thr, with slow tight binding kinetics^20^,^32^. However, it is appreciated that slow, tight binding active site-directed inhibitors can form weak encounter complexes that are readily competed by reaction substrates, before isomerizing to a high-affinity non-productive state that may be distinct from that favoured by substrates such as _L-_Thr and ATP^33^. This behaviour will generate a noncompetitive kinetic pattern for an inhibitor that behaves competitively in functional assays^34^.

**Figure 4:**
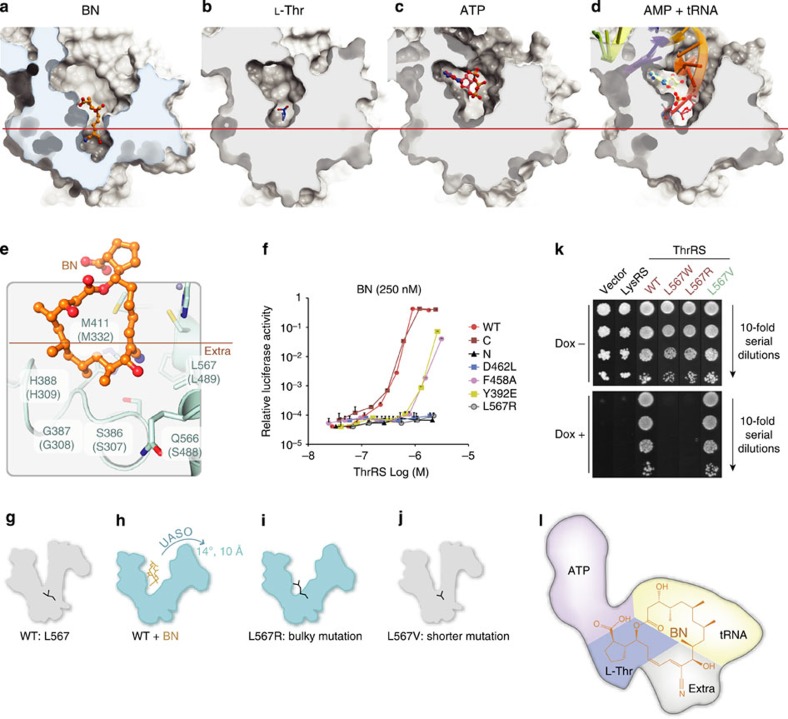
Critical regulation of ThrRS conformation and activity through the extra binding site of BN. (**a**–**d**) Binding cavity of ThrRS–BN complex in comparison with those of ThrRSs in complex with _L-_Thr (PDB: 1EVK), ATP (PDB: 1NYR) and AMP+tRNA^Thr^ (PDB: 1QF6). The approximate _L-_Thr pocket boundary is shown as a red line with bound ligands as sticks. BN binds 4 Å deeper and induces a cavity ~8 Å below the bottom of _L-_Thr pocket. (**e**) Extra hydrophobic pocket occupied by BN (orange). Interacting residues are shown as sticks. (**f**) In addition to human ThrRS full-length (WT) and fragments (*N*: 1–322; C: 322–723), four indicated mutants were tested to rescue the RRL-based *in vitro* protein translation inhibited by 250 nM BN. D462L misses one hydrogen bond with the hydroxyl group of _L-_Thr; F458A misses the stacking interaction with the adenine group of ATP; Y392E causes repulsion to the tRNA backbone phosphate group and L567R fills the space of the fourth subsites. (**g**–**j**) Schematic diagrams of the conformational change in ThrRS. The BN-induced upper active site opening (UASO) is denoted, whereas BN is shown as orange lines. Bulky space-filling mutation (L567R) and shorter mutation (L567V) are shown as black lines. (**k**) Yeast ThrRS (yTHS1) was replaced by human wide-type ThrRS or mutants in supporting yeast growth. Empty vector and human lysyl-tRNA synthetase (LysRS) are used as control. The expression of endogenous yeast ThrRS was switched off by the addition of doxycycline (Dox) to the yeast growth medium. Tenfold serial dilutions of freshly gown yeast cells were spotted onto selective media synthetic complete medium without histodine (SCM-HIS) containing 2% galactose with or without Dox. (**l**) Schematic map showing BN occupied four sites on ThrRS: _L_-Thr site, ATP site, tRNA site and an extra site. Each site inhibits the translational activity of ThrRS.

We investigated whether BN induces such a state by comparing the *E. coli* ThrRS–BN structures with those of canonical ligands: _L-_Thr^26^, ATP^35^ and AMP together with tRNA^27^. By comparison with all three standard aminoacylation substrates, _L-_Thr, ATP, and AMP+tRNA^Thr^ (Fig. 4b–d), BN is inserted 4 Å deeper into the active site cleft (Fig. 4a). The additional interactions associated with the deeper binding lead to a significant conformational opening at the upper side of active site, thus highlighting an induced-fit component to the binding mechanism of BN (Supplementary Fig. 10). This upper active site opening also distinguishes the ThrRS–BN complex from the comparatively modest conformational change observed when ThrRS is bound to its aminoacylation substrates (Supplementary Fig. 10a). Prior fluorescence observations suggested that a strong conformational change of ThrRS occurs upon binding to BN^20^,^36^. In our structures, a major feature of the BN-induced conformational change is a 14° rotation of the α helix comprised by residues 431–448 that produce a 10-Å displacement of the preceding loop (Supplementary Fig. 10b). This structural change is observed in both the human and *E. coli* ThrRS–BN complexes (Supplementary Fig. 3b).

In addition to the segments of the polyketide ring (C15-C3) that overlap with the canonical substrate-binding sites, additional portions of the BN polyketide ring project into the back of the active site cavity, effectively creating a fourth BN-binding subsite (Fig. 2b–e and Fig. 4e). This fourth BN-binding site consists of six highly conserved residues in bacterial and eukaryotic ThrRSs that constitute the back of the _L_-Thr pocket and have minimal exposure to solvent. This extra site provides interactions with the C4–C14 moeity of the polyketide ring, which represents a majority of carbons in the 18-membered macrolide ring (Fig. 4e). Notably, the 12′ cyano moiety, which strongly contributes to BN potency^37^, forms van der Waals contacts with the residues C413, L567, H388. The nearby 10′-OH group is hydrogen-bound to D564, representing the only hydrophilic interaction in this binding site. The rest of the macrolide is enveloped by hydrophobic contacts with the floor of the fourth subsite, principally contributed by S386, G387, H388, H391 and Y392. Interestingly, when ThrRS is not bound by BN, residues of this extra site are packed inside ThrRS forming a hydrophobic cluster (Fig. 4b–d). These residues are less conserved than those of the three substrate-binding sites (Supplementary Fig. 5). With the exception of C413 and D564, which are used for _L-_Thr recognition, all others are highly variable in archaeal ThrRS, and may contribute to the resistance of archaeal ThrRS to BN (Supplementary Figs 5 and 8). In summary, this fourth BN-binding subsite is distinguished from the aminoacylation substrate subsites by its induced-fit character, which arises from the unique manner in which the macrolide structure is inserted into the distal portion of the ThrRS active site cavity.

### The extra binding site redundantly regulates ThrRS activity

These observations raised the question of whether the extra BN-binding subsite regulates the activity of ThrRS to the degree seen with the canonical substrate sites. To investigate this hypothesis, various binding site mutants were tested for their ability to rescue translation in rabbit reticulocyte lysates incubated with inhibitory concentrations of BN. Several mutants (D462L, F458A and Y392E) that perturb key contacts with aminoacylation substrates were employed as positive controls, along with a construct consisting of the first 322 amino acids of ThrRS (1–322). All four controls were dramatically reduced in their ability to rescue BN-dependent inhibition of translation, indicating the significant loss of aminoacylation activities (Fig. 4f). Notably, the D462L mutant failed to rescue the translation at any tested concentration, indicating that it lost both the aminoacylation and BN-binding abilities (Fig. 4f). L567 is located at the bottom of the fourth subsite, and its side chain forms direct contacts to C11-C12 of BN (Fig. 4e). As observed with D462L, L567R failed to rescue translation at all concentrations (Fig. 4f).

To further confirm the importance of the extra binding site for translation in cell, we used an *in vivo* yeast complementary survival assay. To probe the consequences of filling the extra BN-binding site, L567 was substituted with the bulkier tryptophan and arginine side chains, generating the mutants L567W and L567R (Fig. 4g–i). Both mutants completely lost the rescuing activity (Fig. 4k). The effect of leaving the fourth site less occupied was tested by examining the phenotype of L567V ThrRS, which removes one methyl group from this key side chain (Fig. 4j). Earlier work investigating the properties of L567V showed that its *K*_i_ for the BN-related derivative BC194 is increased eightfold relative to wild type^8^. Indeed, L567V fully rescued the yeast strain (Fig. 4k). Thus, although the L567V substitution affects a key interaction with BN, it leaves aminoacylation and protein synthesis unaffected. Therefore, these results revealed that the filling of the induced extra space by BN redundantly blocks the aminoacylation activity of ThrRS, making the BN a novel potent, quadrivalent inhibitor for translation (Fig. 4l).

## Discussion

Collectively, these results represent a step towards implementation of small molecule based specific regulation of the AARSs. After 48 years of investigation of its inhibition of cellular function^4^, BN is now shown to be distinguished from other small molecules that inhibit the canonical function of AARSs, and that mimic the structures of substrates of AARSs.

Because of their important functions, inhibitors of AARSs have been used as medicine or rigorously tested in clinical trials for therapeutic applications in microbial infections, cancers and autoimmune diseases^38^,^39^,^40^,^41^,^42^,^43^. For example, the natural product mupirocin inhibits isoleucyl-tRNA synthetase activity and is approved as a topical treatment for bacterial skin infections. Febrifugine is the active component of the Chinese herb Chang Shan (*Dichroa febrifuga Lour*.), which has been used for treating malaria-induced fever for about 2,000 years. Halofuginone (HF), the halogenated derivative of febrifugine, has been tested in clinical trials for potential therapeutic applications in cancer and fibrotic disease^44^. Recently, HF was reported to inhibit Th17 cell differentiation by activating the amino-acid response pathway, via inhibition of the aminoacylation function of human prolyl-tRNA synthetase^38^,^45^. The recently developed inhibitor AN2690 binds to fungal leucyl-tRNA synthetase and is licensed for the treatment of onychomycosis^46^. These examples highlight the tremendous potential of AARS inhibitors in medical applications, and illustrate how knowledge of the inhibition mechanism can promote the development of novel therapies.

**Figure 5:**
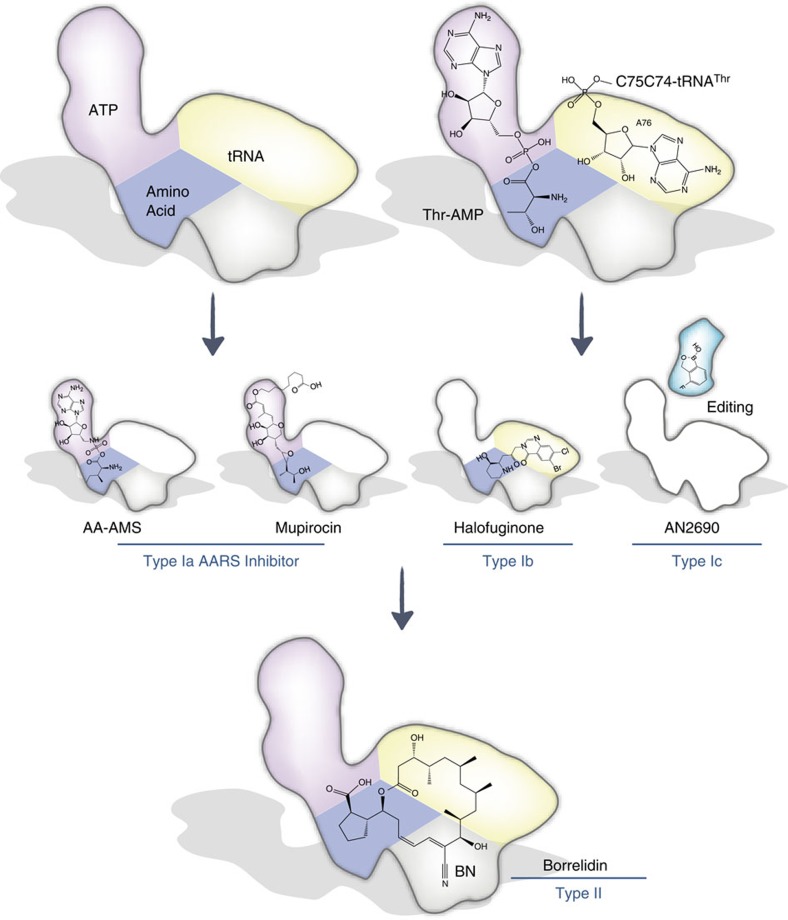
BN represents a new class of AARS inhibitor. All aminoacyl-tRNA synthetases (AARSs) contain three pockets for binding its natural substrates (amino acid, ATP and tRNA-A76) to catalyse the two-step aminoacylation reaction. It involves the activation of amino acid by ATP to form an aminoacyl adenylate (AA-AMP), followed by a transfer of the aminoacyl-group from the high-energy intermediate AA-AMP to the 3′-OH of tRNA. Type Ia inhibitors including AA-AMS, Agrocin 84, mupirocin *etc.*, mimic AA-AMP and occupy amino acid and ATP pockets (see also Table 1). The type Ib inhibitor halofuginone (HF) mimics prolyl-tRNA 3′-A76, and binds to proline and tRNA-A76 pockets on prolyl-tRNA synthetase (ProRS). The type Ic inhibitor AN2690 co-binds and traps tRNA in the leucyl-tRNA synthetase (LeuRS)-editing domain. The new (type II) inhibitor Borrelidin (BN) binds at a joint region of amino acid, ATP and tRNA pockets, as well as an extra induced-fit pocket in the active site cavity of ThrRS. As an 18-member macrolide, BN does not resemble the native substrates, but binds to the active site of ThrRS through an unseen geometrically fitting mechanism.

Three classes of inhibition have been found for AARS inhibitors (Table 1). The most common compounds (Type Ia) either mimic the reaction intermediate AA-AMP (AA-AMS)^29^ or occupy the AA-AMP-binding site (mupirocin, for isoleucyl-tRNA synthetase)^40^. HF functions by binding prolyl-tRNA synthetase in an ATP-dependent way and mimics both the amino acid and tRNA 3′-A76 (Type Ib)^38^,^41^. AN2690 inhibits fungal leucyl-tRNA synthetase by binding to the editing site and covalently immobilizing the 3′ A76 nucleotide of tRNA (Type Ic)^39^. In contrast, our structural and experimental data indicate that BN binds to all three key substrate-binding subsites in the ThrRS enzymatic pocket, physically excluding all three physiological ThrRS substrates. Inhibition of translation by BN can be rescued by the addition of each substrate, indicating that BN functions as a triple-competitive inhibitor. Despite its absence of structural resemblance to any substrate and lack of chemically similar interactions, the macrolide ring of BN extends into each of the core substrate-binding subsites of the ThrRS active site cavity, thereby defining a new class (Type II) of AARS inhibitor (Fig. 5).

In addition to the simultaneous competition with all three substrates of ThrRS, the complex structures indicate that BN wedges open the floor of the ThrRS active site and induces an extra binding site by virtue of its unique macrolide structure. This fourth ‘orthogonal’ site does not overlap with the substrate-binding sites and is formed by mostly hydrophobic residues that close up the cavity in the absence of BN (Fig. 4e,i). Owing to their role in forming inner core packing interactions of the enzyme, these hydrophobic residues are less conserved than other residues forming the catalytic cavity. Most alterations of the BN-binding residues in archaeal ThrRSs are located in this fourth site (Supplementary Fig. 8). Interestingly, BN-resistant mutations (L489 in *E. coli*) identified in survival screens are also found to be at the fourth site (Supplementary Fig. 7d). The ThrRS of BN-producing and self-resistant bacteria, *Streptomyces parvulus,* only has 4 out of the 18 key residues (Y334, H488, Y515 and T516) unconserved from the other BN-sensitive bacteria. These residues correspond to H391, F539, Q566 and L567 in human. All of them are located in the fourth site, and oriented to potentially clash with BN in our structure model (Supplementary Fig. 7b). The observation that many substitutions associated with BN resistance map to the fourth BN-binding pocket suggests that it serves as a key site for the kingdom-wide resistance to BN and a hotspot for developing BN resistance (Supplementary Fig. 8). In fact, our results indicate that simply filling the extra site space (L567R) is sufficient to inactivate aminoacylation of ThrRS (Fig. 4f,k) and inhibits its function for protein synthesis. Thus, this fourth BN-binding site further defines BN as a quadrivalent inhibitor that redundantly blocks protein translation through ThrRS.

With an IC_50_ of 0.8–7 nM against ThrRS function, BN represents one of the most potent of all known AARS inhibitors (Table 1). Recent studies reveal that several clinically significant drug–enzyme complexes are similarly high-affinity active site-directed inhibitors that compete with substrates and isomerize the target to a non-productive conformation^34^. These results raise the possibility that additional compounds like BN with no structural similarity to amino acids, ATP and tRNAs might be developed to modulate the functions of other AARSs. By visualizing the modular nature of ThrRS-specific recognition by BN, this study may facilitate the rational design of additional novel active site-binding reagents with a range of useful applications.

## Methods

### Protein preparation

The N-terminal His-tagged human ThrRS (residues 322–723) was constructed in a pET28a vector. The protein was expressed in strain BL21 (DE3) and induced with 0.2 mM isopropyl-β-D-thiogalactoside for 20 h at 16 °C. The cell pellet (from 4 to 8 litres) was lysed in NTA-wash buffer (500 mM NaCl, 20 mM Tris-HCl, pH 8.0, 30 mM imidazole), loaded onto a Ni-HiTrap column and washed with NTA-wash buffer. The protein was eluted with NTA-elution buffer (500 mM NaCl, 20 mM Tris-HCl, pH 8.0, 250 mM imidazole). The eluted protein was further purified by a QAE anion exchange column with NaCl gradient. The peak fractions of the protein were then concentrated for crystallization. The N-terminal His-tagged *E. coli* ThrRS (aa242–642), and the full-length N-terminal His-tagged human ThrRSs (aa1–723) were also prepared similarly.

### Crystallization and structure determination

Crystallizations were all done by the sitting drop method. To crystallize human ThrRS–BN complex, protein solution (10 mg ml^−1^) was pre-mixed with 2 mM BN (BN) at 4 °C. The protein was then crystallized by mixing 0.5 μl of protein solution with 0.5 μl of precipitant solution, containing 0.1 M calcium acetate, 10% PEG4000 and 0.1 M sodium acetate, pH 4.5. After incubation at 18 °C for 3–7 days, the crystals were flash-frozen in liquid nitrogen for data collection with a cryo solution consisting of 0.8 M calcium acetate, 8% PEG4000, 0.8 M sodium acetate, pH 4.5, and 20% glycerol. Diffraction data were obtained from the LS-CAT 21-ID-G beamline at the Advanced Photon Source (APS).

To crystallize *E. coli* ThrRS–BN complex, protein solution (30 mg ml^−1^) was pre-mixed with 2 mM BN at 4 °C with/without 5 mM _L-_Thr, 5 mM AMPcPP and 10 mM MgCl_2_. The protein was then crystallized by mixing 0.5 μl of protein solution with 0.5 μl of precipitant solution, containing 15% PEG400, 0.1 M MES, pH6.5. After incubation at 18 °C for 3–7 days, the resulting crystals were flash-frozen in liquid nitrogen for data collection using a cryo solution containing 12% PEG400, 0.8 M MES, pH 6.5, and 20% glycerol. Data sets were obtained from beamline 7-1 at the Stanford Synchrotron Radiation Lightsource (SSRL).

All data sets were integrated and scaled with HKL2000 (ref. 47). The structures were determined by molecular replacement based on the *E. coli* ThrRS structure (PDB: 1EVK) in programme Molrep^48^. After corrections for bulk solvent and overall *B* values, data were refined by iterative cycles of positional refinement and TLS refinement with PHENIX^49^ and model building with COOT^50^. All current models have good geometry and no residues are in the disallowed region of the Ramachandran plot. Data collection and model statistics are given in Table 2.

### Aminoacylation assay with BN

The aminoacylation assay was carried out in a buffer containing 50 mM HEPES-KOH (pH 7.6), 25 mM KCl, 10 mM MgCl_2_, 5 mM ATP, 2 mg ml^−1^ yeast tRNA, 10 μM [^3^H] _L-_Thr, 0.5–10 nM BN (dissolved in dimethylsulphoxide) and 100 nM of human ThrRS. Reactions were initiated with enzyme and conducted in a 37 °C heat block. After 20 min, reactions were quenched on Whatman filter pads that were presoaked with 5% trichloroacetic acid. The pads were washed three times for 10 min each with cold 5% trichloroacetic acid, once with cold 100% ethanol. The washed pads were then dried. Radioactivity was quantified in a scintillation counter (Beckman Coulter).

### *In vitro* translation assay

The effects of BN and added amino acids on cell-free protein translation were assayed in a rabbit reticulocyte lysate according to the manufacturer’s instructions (Promega), with the exception that no extra amino-acid mix was added in the assays. Firefly luciferase mRNA of 0.02 mg ml^−1^ was used in all the translation assays. BN of 250 nM was used in the amino acids-rescue assay. To increase the sensitivity to observe the rescuing effect of ATP and tRNA^Thr^ on BN inhibition, the original rabbit reticulocyte lysate was diluted by tenfold with buffer A and T, respectively. Buffer A contains 10 μg ml^−1^ yeast total tRNA, 80 mM KCl, 0.25 mM MgCl_2_, 0.1 mM spermidine and 50 μM amino-acid mixture. Buffer T contains 80 mM KCl, 0.25 mM MgCl_2_ and 0.1 mM spermidine. The incubation time was increased from 1.5 to 20 h, and everything else was kept the same as non-diluted system.

### Yeast viability assay

The translation activities of different human ThrRS mutants were checked in the yeast viability assay, when the yeast ThrRS was replaced with human ThrRS proteins. The cDNA sequences encoding full-length human ThrRS and its mutations were constructed in the p413-GAL1 vector multi-cloning site. The plasmids were then transformed into the yeast tet-promoters Hughes collection (yTHC) mutant strain obtained from Open Biosystems according to the LiAc/ssDNA/PEG method. yTHC was provided in the haploid strain R1158 background (URA: CMV-tTA MATa his3-1 leu2-0 met15-0). The endogenous promoter of yeast cytoplasmic ThrRS gene (*ths1*) has been replaced with a TET-titratable promoter in the yTHC genome. Thus, the expression of the gene can be switched off by the addition of doxycycline to the yeast’s growth medium. Tenfold serial dilutions of freshly grown yeast cells were spotted onto selective media synthetic complete medium without histodine containing 2% galactose (with or without doxycycline). Plates were incubated at 30 °C for 4 days and then photographed.

## Author contributions

P.F., X.Y., S.K., C.S.F. and M.G. designed all experiments. P.F., X.Y., S.J., A.M., K.C., X.C. and M.G. performed the experiments. All authors analysed the data and contributed to manuscript preparation. P.F., X.Y., C.S.F. and M.G. wrote the manuscript.

## Additional information

**Accession codes.** The atomic coordinates and structure factors of human ThrRS–BN and the two *E. coli* ThrRS–BN complexes have been deposited in the Protein Data Bank (PDB) with the accession codes 4P3N, 4P3O and 4P3P, respectively.

**How to cite this article:** Fang, P. *et al.* Structural basis for full-spectrum inhibition of translational functions on a tRNA synthetase. *Nat. Commun.* 6:6402 doi: 10.1038/ncomms7402 (2015).

## Supplementary Material

Supplementary Information Supplementary Figures 1-10 and Supplementary References

Supplementary Movie 1 This movie shows the detailed binding of BN to human and E. coli ThrRS. Clearly seen in how tightly bound BN sterically clashes with all 3 substrates of ThrRS: L-Thr, ATP and tRNA. The renderings used 6 structures analyzed in this work (PDB: 4P3N, 4P3O, 4P3P, 1EVK, 1NYR, 1QF6). This movie is prepared with ICM molecular modeling suite (Molsoft LLC) and PyMOL (www.pymol.org).

## Figures and Tables

**Table 1:** List of characterized AARS inhibitors.

**Categories**	**Inhibitors**	***K***_**i**_**/IC**_**50**_**/Kd**	**Activities**
Type Ia:Mimetics binding amino acid and/or ATP-binding pocket	Mupirocin—IleRS	2.5–32 nM	*E. coli*, yeast, *Ps.fluorescens*, rat, IleRS ATP/[32P]-pyrophosphate exchange (ATP-PPi exchange) and aminoacylation^51^,^52^*B. subtilis and S. aureus* IleRS mupirocin uptake^53^
	Ile-ol-AMP	30 nM	*S. aureus* IleRS stop-flow fluorescence^54^
	Thr-AMS–ThrRS	~13 nM	*E. coli* and human ThrRS ATP-PPi exchange^55^
	10a–ThrRS	~3 nM	*E. coli* and human ThrRS ATP-PPi exchange^55^
	Cladosporin–LysRS	40–90 nM	Plasmodium parasites growth^56^
		74.3 μM	Human Hela cell growth^56^
	Indolmycin–TrpRS	160 nM	*S. aureus* Tryptophan uptake^57^
	Agrocin 84–LeuRS	<10 nM	*A. tumefaciens* LeuRS aminoacylation^58^
	SB217452–SerRS	8 nM	*S. aureus* and rat SerRS aminoacylation^59^
Type Ib:Mimetics binding amino acid and tRNA-binding pockets	HF–ProRS	2.5–18.3 nM	T-cell proliferation in response to alloantigen or IL-2 (ref. 60);*P. berghei* sporozoite load in HepG2 cells^61^;Human ProRS aminoacylation^45^
Type Ic:Trapping tRNA at editing site	AN2690–LeuRS	1.85 μM	Yeast LeuRS aminoacylation^39^
	ZCL039–LeuRS	1.73 μM	*S. pneumoniae* LeuRS aminoacylation^62^
Type II:Non-mimetic, blocking all substrate binding by geometrical fitting	BN–ThrRS	0.8–7 nM	Rat aorta tube formation^11^;Plasmodium growth^10^;*E. coli* and *S. solfataricus* ThrRS ATP-PPi exchange^20^Human ThrRS aminoacylation (*this study*)

AARS, aminoacyl-tRNA synthetase; BN, Borrelidin; HF, Halofuginone; IleRS, isoleucyl-tRNA synthetase; LeuRS, leucyl-tRNA synthetase; LysRS, lysyl-tRNA synthetase; ProRS, prolyl-tRNA synthetase; SerRS, seryl-tRNA synthetase; ThrRS, threonyl-tRNA synthetase.

**Table 2:** Crystallographic statistics of ThrRS–BN complex structures.

	**Human ThrRS-BN**	***E. coli*** ThrRS-BN 1*	***E. coli*** **ThrRS-BN 2**†
*Data collection*			
Space group	*P1*	*P2*_*1*_*2*_*1*_*2*_*1*_	*P2*_*1*_*2*_*1*_*2*_*1*_
Cell dimensions			
*a*, *b*, *c* (Å)	63.87, 78.00, 118.05	94.53, 107.64, 109.62	94.45, 107.62, 109.21
*α*, *β*, *γ* (°)	86.99, 83.32, 84.39	90, 90, 90	90, 90, 90
Resolution (Å)	50.00–2.60 (2.74–2.60)‡	50–2.10 (2.18–2.10)	50–2.50 (2.59–2.50)
*R*_sym_ or *R*_merge_ (%)	9.6 (33.2)	11.8 (75.0)	10.5 (51.5)
*I*/s*I*	5.8 (2.0)	15.5 (2.6)	15.1 (2.8)
Completeness (%)	98.1 (97.5)	99.1 (99.8)	94.1 (88.9)
Redundancy	2.2 (2.2)	6.6 (6.5)	5.9 (5.2)
			
*Refinement*			
Resolution (Å)	50.00–2.60 (2.69–2.60)	50–2.10 (2.16–2.10)	50–2.50 (2.59–2.50)
No. of reflections	67,853 (6794)	63,912 (4564)	34,852 (2457)
*R*_work_/*R*_free_ (%)	22.8/25.5	20.0/21.9	19.8 (22.4)
No. of atoms			
Protein	12,973	6,536	6,536
Chain A	3,260	3,262	3,262
Chain B	3,253	3,274	3,274
Chain C	3,249	—	—
Chain D	3,211	—	—
Zn^2+^	4	2	2
BN	140	70	70
Solvent	390	656	381

B*-factors (Å*^*2*^)			
Protein	56.38	24.8	35.8
Chain A	52.40	25.30	36.41
Chain B	53.85	24.29	35.24
Chain C	59.82	—	—
Chain D	59.51	—	—
Zn^2+^	51.41	19.84	28.59
BN	48.78	17.36	30.03
Solvent	52.10	34.75	38.96

Root mean square deviations			
Bond lengths (Å)	0.008	0.012	0.007
Bond angles (°)	1.027	1.220	1.470
Ramachandran plot			
Most favoured (%)	98.2	98.0	98.0
Additional allowed (%)	1.8	2.0	2.0

BN, Borrelidin; ThrRS, threonyl-tRNA synthetase.

^*^This crystal was co-crystallized with BN in the presence of threonine, AMPcPP and MgCl_2_. No electric density of these substrates was observed.

^†^The crystal was co-crystallized with BN without the presence of threonine, AMPcPP or MgCl_2_.

^‡^Values in parentheses are for highest-resolution shell.

## References

[b1] Dickinson L., Griffiths A. J., Mason C. G. & Mills R. F. Anti-viral activity of two antibiotics isolated from a species of Streptomyces. Nature 206, 265–268 (1965) . 4284424 10.1038/206265a0

[b2] Liu C. X. *et al.* Antifungal activity of borrelidin produced by a Streptomyces strain isolated from soybean. J. Agric. Food Chem. 60, 1251–1257 (2012) . 22242825 10.1021/jf2044982

[b3] Olano C. *et al.* Biosynthesis of the angiogenesis inhibitor borrelidin by Streptomyces parvulus Tu4055: insights into nitrile formation. Mol. Microbiol. 52, 1745–1756 (2004) . 15186422 10.1111/j.1365-2958.2004.04090.x

[b4] Hutter R., Poralla K., Zachau H. G. & Zahner H. [Metabolic products of microorganisms. 51. On the mechanism of action of borrelidin-inhibition of the threonine incorporation in sRNA]. Biochem. Z 344, 190–196 (1966) . 4860826

[b5] Nass G. & Hasenbank R. Effect of Borrelidin on the threonyl-tRNA-synthetase activity and the regulation of threonine-biosynthetic enzymes in *Saccharomyces cerivisiae*. Mol. Gen. Genet. 108, 28–32 (1970) . 5472935 10.1007/BF00343181

[b6] Sugawara A. *et al.* Borrelidin analogues with antimalarial activity: design, synthesis and biological evaluation against *Plasmodium falciparum* parasites. Bioorg. Med. Chem. Lett. 23, 2302–2305 (2013) . 23499502 10.1016/j.bmcl.2013.02.075

[b7] Dorgerrloh M. *et al.* Borrelidin insecticide and herbicide and its peparation by fermentation. Ger Offen 11,, DE 3607287 (1988) .

[b8] Williams T. F., Mirando A. C., Wilkinson B., Francklyn C. S. & Lounsbury K. M. Secreted Threonyl-tRNA synthetase stimulates endothelial cell migration and angiogenesis. Sci. Rep. 3, 1317 (2013) . 23425968 10.1038/srep01317 PMC3578223

[b9] Novoa E. M. *et al.* Analogs of natural aminoacyl-tRNA synthetase inhibitors clear malaria in vivo. Proc. Natl Acad. Sci. USA 111, E5508–E5517 (2014) . 25489076 10.1073/pnas.1405994111 PMC4280603

[b10] Otoguro K. *et al.* *In vitro* and *in vivo* antimalarial activities of a non-glycosidic 18-membered macrolide antibiotic, borrelidin, against drug-resistant strains of Plasmodia. J. Antibiot. (Tokyo) 56, 727–729 (2003) . 14563165 10.7164/antibiotics.56.727

[b11] Wakabayashi T. *et al.* Borrelidin is an angiogenesis inhibitor; disruption of angiogenic capillary vessels in a rat aorta matrix culture model. J. Antibiot. (Tokyo) 50, 671–676 (1997) . 9315080 10.7164/antibiotics.50.671

[b12] Funahashi Y. *et al.* Establishment of a quantitative mouse dorsal air sac model and its application to evaluate a new angiogenesis inhibitor. Oncol. Res. 11, 319–329 (1999) . 10757446

[b13] Poralla K. & Zahner H. [Metabolic products of microorganisms. 62. The inhibition of the incorporation of threonine in sRNA in cell-free systems and of the synthesis of proteins and nucleic acids in the cell by the antibiotic borrelidin]. Arch. Mikrobiol. 61, 143–153 (1968) . 4974079

[b14] Nass G. & Thomale J. Alteration of structure of level of threonyl-tRNA-synthetase in Borrelidin resistant mutants of E. coli. FEBS Lett. 39, 182–186 (1974) . 4368395 10.1016/0014-5793(74)80046-3

[b15] Nass G. & Poralla K. Genetics of borrelidin resistant mutants of Saccharomyces cerivisiae and properties of their threonyl-tRNA-synthetase. Mol. Gen. Genet. 147, 39–43 (1976) . 785224 10.1007/BF00337933

[b16] Gantt J. S., Bennett C. A. & Arfin S. M. Increased levels of threonyl-tRNA synthetase in a borrelidin-resistant Chinese hamster ovary cell line. Proc. Natl Acad. Sci. USA 78, 5367–5370 (1981) . 6946478 10.1073/pnas.78.9.5367 PMC348746

[b17] Carter C. W. Jr. Cognition, mechanism, and evolutionary relationships in aminoacyl-tRNA synthetases. Annu. Rev. Biochem. 62, 715–748 (1993) . 8352600 10.1146/annurev.bi.62.070193.003435

[b18] Ibba M. & Soll D. Aminoacyl-tRNA synthesis. Annu. Rev. Biochem. 69, 617–650 (2000) . 10966471 10.1146/annurev.biochem.69.1.617

[b19] Habibi D. *et al.* Borrelidin, a small molecule nitrile-containing macrolide inhibitor of threonyl-tRNA synthetase, is a potent inducer of apoptosis in acute lymphoblastic leukemia. Invest. New Drugs 30, 1361–1370 (2012) . 21678129 10.1007/s10637-011-9700-y

[b20] Ruan B. *et al.* A unique hydrophobic cluster near the active site contributes to differences in borrelidin inhibition among threonyl-tRNA synthetases. J. Biol. Chem. 280, 571–577 (2005) . 15507440 10.1074/jbc.M411039200

[b21] Francklyn C., Musier-Forsyth K. & Martinis S. A. Aminoacyl-tRNA synthetases in biology and disease: new evidence for structural and functional diversity in an ancient family of enzymes. RNA 3, 954–960 (1997) . 9292495 PMC1369542

[b22] Cusack S. Aminoacyl-tRNA synthetases. Curr. Opin. Struct. Biol. 7, 881–889 (1997) . 9434910 10.1016/s0959-440x(97)80161-3

[b23] Woese C. R., Olsen G. J., Ibba M. & Soll D. Aminoacyl-tRNA synthetases, the genetic code, and the evolutionary process. Microbiol. Mol. Biol. Rev. 64, 202–236 (2000) . 10704480 10.1128/mmbr.64.1.202-236.2000 PMC98992

[b24] Rigden D. J. Archaea recruited D-Tyr-tRNATyr deacylase for editing in Thr-tRNA synthetase. RNA 10, 1845–1851 (2004) . 15525705 10.1261/rna.7115404 PMC1370672

[b25] Ibba M. & Soll D. Aminoacyl-tRNAs: setting the limits of the genetic code. Genes Dev. 18, 731–738 (2004) . 15082526 10.1101/gad.1187404

[b26] Sankaranarayanan R. *et al.* Zinc ion mediated amino acid discrimination by threonyl-tRNA synthetase. Nat. Struct. Biol. 7, 461–465 (2000) . 10881191 10.1038/75856

[b27] Sankaranarayanan R. *et al.* The structure of threonyl-tRNA synthetase-tRNA(Thr) complex enlightens its repressor activity and reveals an essential zinc ion in the active site. Cell 97, 371–381 (1999) . 10319817 10.1016/s0092-8674(00)80746-1

[b28] Moras D. Structural and functional relationships between aminoacyl-tRNA synthetases. Trends Biochem. Sci. 17, 159–164 (1992) . 1585461 10.1016/0968-0004(92)90326-5

[b29] Belrhali H. *et al.* Crystal structures at 2.5 angstrom resolution of seryl-tRNA synthetase complexed with two analogs of seryl adenylate. Science 263, 1432–1436 (1994) . 8128224 10.1126/science.8128224

[b30] Pelham H. R. & Jackson R. J. An efficient mRNA-dependent translation system from reticulocyte lysates. Eur. J. Biochem. 67, 247–256 (1976) . 823012 10.1111/j.1432-1033.1976.tb10656.x

[b31] Kawamura T. *et al.* Anti-angiogenesis effects of borrelidin are mediated through distinct pathways: threonyl-tRNA synthetase and caspases are independently involved in suppression of proliferation and induction of apoptosis in endothelial cells. J. Antibiot. (Tokyo) 56, 709–715 (2003) . 14563161 10.7164/antibiotics.56.709

[b32] Paetz W. & Nass G. Biochemical and immunological characterization of threonyl-tRNA synthetase of two borrelidin-resistant mutants of *Escherichia coli* K12. Eur. J. Biochem. 35, 331–337 (1973) . 4577856 10.1111/j.1432-1033.1973.tb02843.x

[b33] Copeland R. A. in Evaluation of Enzyme Inhibitors in Drug Discovery: A Guide for Medicinal Chemists and Pharmacologists 2nd edn 203–244John Wiley and Sons (2013) . 16350889

[b34] Blat Y. Non-competitive inhibition by active site binders. Chem. Biol. Drug Des. 75, 535–540 (2010) . 20374252 10.1111/j.1747-0285.2010.00972.x

[b35] Torres-Larios A., Sankaranarayanan R., Rees B., Dock-Bregeon A. C. & Moras D. Conformational movements and cooperativity upon amino acid, ATP and tRNA binding in threonyl-tRNA synthetase. J. Mol. Biol. 331, 201–211 (2003) . 12875846 10.1016/s0022-2836(03)00719-8

[b36] Gao Y. M. *et al.* Borrelidin, a potent antifungal agent: insight into the antifungal mechanism against *Phytophthora sojae*. J. Agric. Food. Chem. 60, 9874–9881 (2012) . 22967236 10.1021/jf302857x

[b37] Schulze C. J. *et al.* Borrelidin B: isolation, biological activity, and implications for nitrile biosynthesis. J. Nat. Prod. 77, 2570–2574 (2014) . 25393949 10.1021/np500727g

[b38] Sundrud M. S. *et al.* Halofuginone inhibits TH17 cell differentiation by activating the amino acid starvation response. Science 324, 1334–1338 (2009) . 19498172 10.1126/science.1172638 PMC2803727

[b39] Rock F. L. *et al.* An antifungal agent inhibits an aminoacyl-tRNA synthetase by trapping tRNA in the editing site. Science 316, 1759–1761 (2007) . 17588934 10.1126/science.1142189

[b40] Silvian L. F., Wang J. & Steitz T. A. Insights into editing from an ile-tRNA synthetase structure with tRNAile and mupirocin. Science 285, 1074–1077 (1999) . 10446055

[b41] Zhou H., Sun L., Yang X. L. & Schimmel P. ATP-directed capture of bioactive herbal-based medicine on human tRNA synthetase. Nature 494, 121–124 (2013) . 23263184 10.1038/nature11774 PMC3569068

[b42] Gadakh B. & Van Aerschot A. Aminoacyl-tRNA synthetase inhibitors as antimicrobial agents: a patent review from 2006 till present. Expert Opin. Ther. Pat. 22, 1453–1465 (2012) . 23062029 10.1517/13543776.2012.732571

[b43] Kim D. G. *et al.* Chemical inhibition of prometastatic lysyl-tRNA synthetase-laminin receptor interaction. Nat. Chem. Biol. 10, 29–34 (2014) . 24212136 10.1038/nchembio.1381 PMC4021855

[b44] Pines M., Snyder D., Yarkoni S. & Nagler A. Halofuginone to treat fibrosis in chronic graft-versus-host disease and scleroderma. Biol. Blood Marrow Transplant. 9, 417–425 (2003) . 12869955 10.1016/s1083-8791(03)00151-4

[b45] Keller T. L. *et al.* Halofuginone and other febrifugine derivatives inhibit prolyl-tRNA synthetase. Nat. Chem. Biol. 8, 311–317 (2012) . 22327401 10.1038/nchembio.790 PMC3281520

[b46] Baker S. J. *et al.* Discovery of a new boron-containing antifungal agent, 5-fluoro-1,3-dihydro-1-hydroxy-2,1- benzoxaborole (AN2690), for the potential treatment of onychomycosis. J. Med. Chem. 49, 4447–4450 (2006) . 16854048 10.1021/jm0603724

[b47] Otwinowski Z. & Minor W. Processing of X-ray diffraction data collected in oscillation mode. Methods Enzymol. 276, 307–326 (1997) . 10.1016/S0076-6879(97)76066-X 27754618

[b48] Vagin A. & Teplyakov A. Molecular replacement with MOLREP. Acta. Crystallogr. D Biol. Crystallogr. 66, 22–25 (2010) . 20057045 10.1107/S0907444909042589

[b49] Adams P. D. *et al.* PHENIX: a comprehensive Python-based system for macromolecular structure solution. Acta. Crystallogr. D Biol. Crystallogr. 66, 213–221 (2010) . 20124702 10.1107/S0907444909052925 PMC2815670

[b50] Emsley P., Lohkamp B., Scott W. G. & Cowtan K. Features and development of Coot. Acta. Crystallogr. D Biol. Crystallogr. 66, 486–501 (2010) . 20383002 10.1107/S0907444910007493 PMC2852313

[b51] Hughes J. & Mellows G. Interaction of pseudomonic acid A with *Escherichia coli* B isoleucyl-tRNA synthetase. Biochem. J. 191, 209–219 (1980) . 6258580 10.1042/bj1910209 PMC1162199

[b52] Racher K. I., Kalmar G. B. & Borgford T. J. Expression and characterization of a recombinant yeast isoleucyl-tRNA synthetase. J. Biol. Chem. 266, 17158–17164 (1991) . 1910039

[b53] Capobianco J. O., Doran C. C. & Goldman R. C. Mechanism of mupirocin transport into sensitive and resistant bacteria. Antimicrob. Agents Chemother. 33, 156–163 (1989) . 2497702 10.1128/aac.33.2.156 PMC171448

[b54] Pope A. J. *et al.* Characterization of isoleucyl-tRNA synthetase from *Staphylococcus aureus*. I: Kinetic mechanism of the substrate activation reaction studied by transient and steady-state techniques. J. Biol. Chem. 273, 31680–31690 (1998) . 9822629 10.1074/jbc.273.48.31680

[b55] Teng M. *et al.* Identification of bacteria-selective threonyl-tRNA synthetase substrate inhibitors by structure-based design. J. Med. Chem. 56, 1748–1760 (2013) . 23362938 10.1021/jm301756m

[b56] Hoepfner D. *et al.* Selective and specific inhibition of the *Plasmodium falciparum* lysyl-tRNA synthetase by the fungal secondary metabolite cladosporin. Cell. Host. Microbe. 11, 654–663 (2012) . 22704625 10.1016/j.chom.2012.04.015 PMC3391680

[b57] Werner R. G. Uptake of indolmycin in gram-positive bacteria. Antimicrob. Agents Chemother. 18, 858–862 (1980) . 7235673 10.1128/aac.18.6.858 PMC352978

[b58] Reader J. S. *et al.* Major biocontrol of plant tumors targets tRNA synthetase. Science 309, 1533 (2005) . 16141066 10.1126/science.1116841

[b59] Stefanska A. L., Fulston M., Houge-Frydrych C. S., Jones J. J. & Warr S. R. A potent seryl tRNA synthetase inhibitor SB-217452 isolated from a Streptomyces species. J. Antibiot. (Tokyo) 53, 1346–1353 (2000) . 11217799 10.7164/antibiotics.53.1346

[b60] Chu T. L., Guan Q., Nguan C. Y. & Du C. Halofuginone suppresses T cell proliferation by blocking proline uptake and inducing cell apoptosis. Int. Immunopharmacol. 16, 414–423 (2013) . 23685128 10.1016/j.intimp.2013.04.031

[b61] Derbyshire E. R., Mazitschek R. & Clardy J. Characterization of Plasmodium liver stage inhibition by halofuginone. ChemMedChem 7, 844–849 (2012) . 22438279 10.1002/cmdc.201200045 PMC3359061

[b62] Hu Q. H. *et al.* Discovery of a potent benzoxaborole-based anti-pneumococcal agent targeting leucyl-tRNA synthetase. Sci. Rep. 3, 2475 (2013) . 23959225 10.1038/srep02475 PMC3747510

