# Supplementary material for: Structural basis for full-spectrum inhibition of translational functions on a tRNA synthetase
Source: Nat Commun. 2015 Mar 31;6:6402. doi: 10.1038/ncomms7402 (PMC4389257; doi:10.1038/ncomms7402)
Supplement: Supplementary Information — Supplementary Figures 1-10 and Supplementary References [file ncomms7402-s1.pdf]

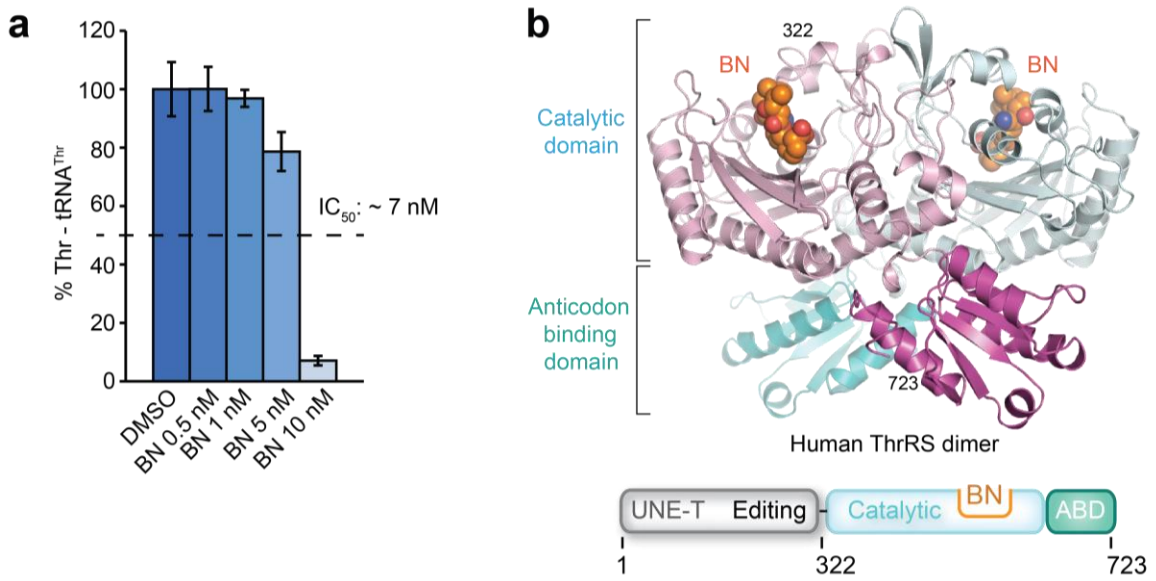

Supplementary Figure 1 | BN binds and inhibits human ThrRS. (a) Aminoacylation activity of recombinant human ThrRS protein was assayed with BN titration (0.5-10 nM), with an estimated IC_{50} of 7 nM. The activity was normalized to DMSO, which is used as the solvent for BN. **(b)** Overview of human ThrRS-BN structure determined at 2.6 Å resolution. Protein is shown in cartoon, and BN (orange) is shown in sphere representation. The crystallized human ThrRS construct (322-723) contains catalytic domain and anticodon binding domain (ABD). ThrRS forms a dimer through its catalytic domain, and each ThrRS monomer binds one BN molecule at the catalytic cavity.

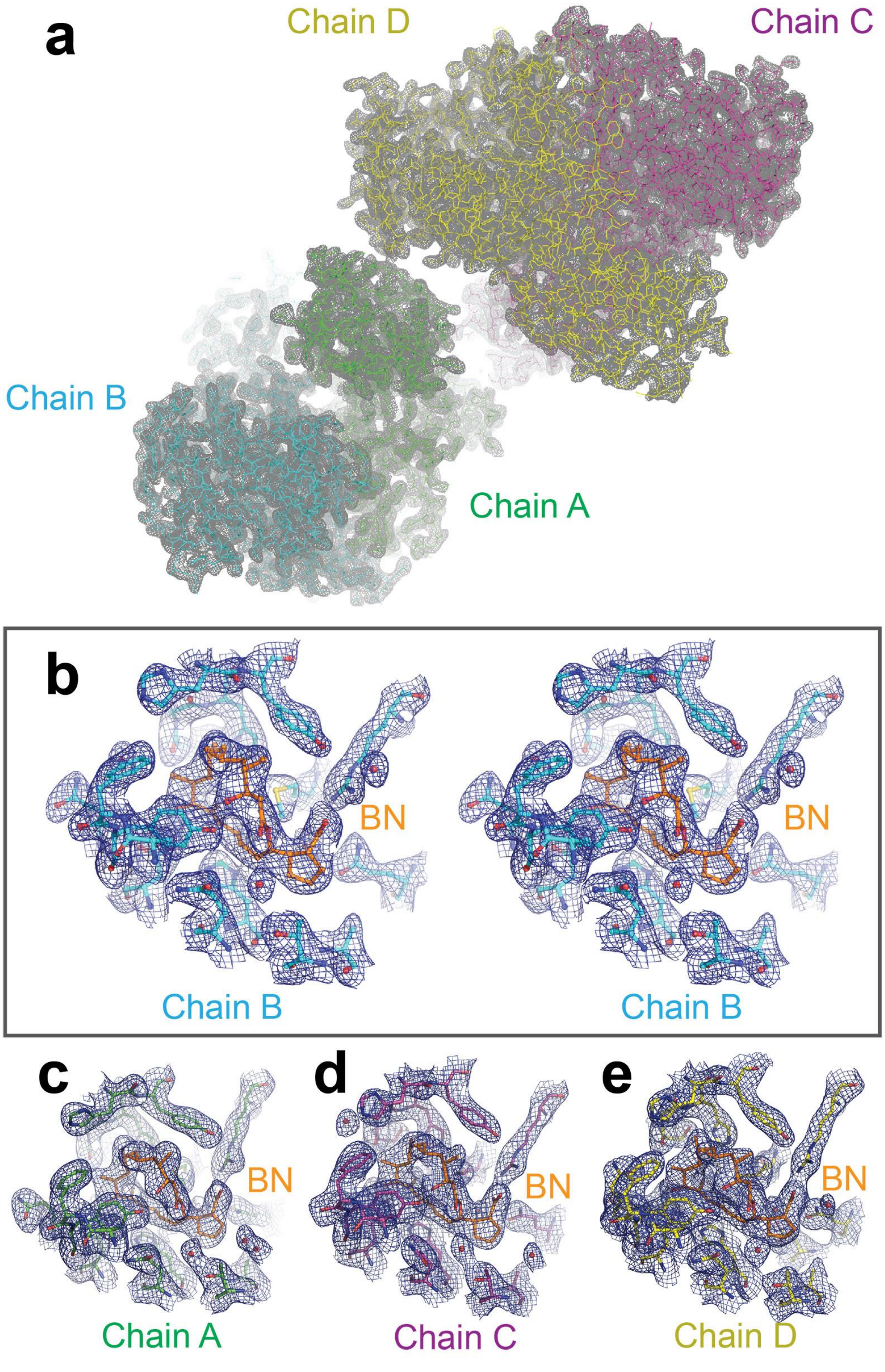
a
Chain D
Chain C
Chain B
Chain A
b
BN
Chain B
BN
Chain B
c
BN
Chain A
d
BN
Chain C
e
BN
Chain D

Supplementary Figure 2 | Electron Density Map of human ThrRS crystal structure. (a) The 2Fo-Fc electron density map of one asymmetric unit, which contains 4 chains, A, B, C, and D. **(b)** Stereo view of the 2Fo-Fc electron density map of BN binding site in chain B, which was used for further analysis. **(c-e)** The 2Fo-Fc electron density map of BN binding site in chain A, C, and D. For **(a-e)**, electron density map is shown as mesh. BN molecules are shown as orange sticks. Protein chains A, B, C, and D are shown as sticks in green, cyan, magenta, and yellow, respectively. Electron densities for all four chains are of good quality. The overall R.M.S.D.s among the four chains are all less than 0.6 Å. All maps were contoured at 1.0 σ.

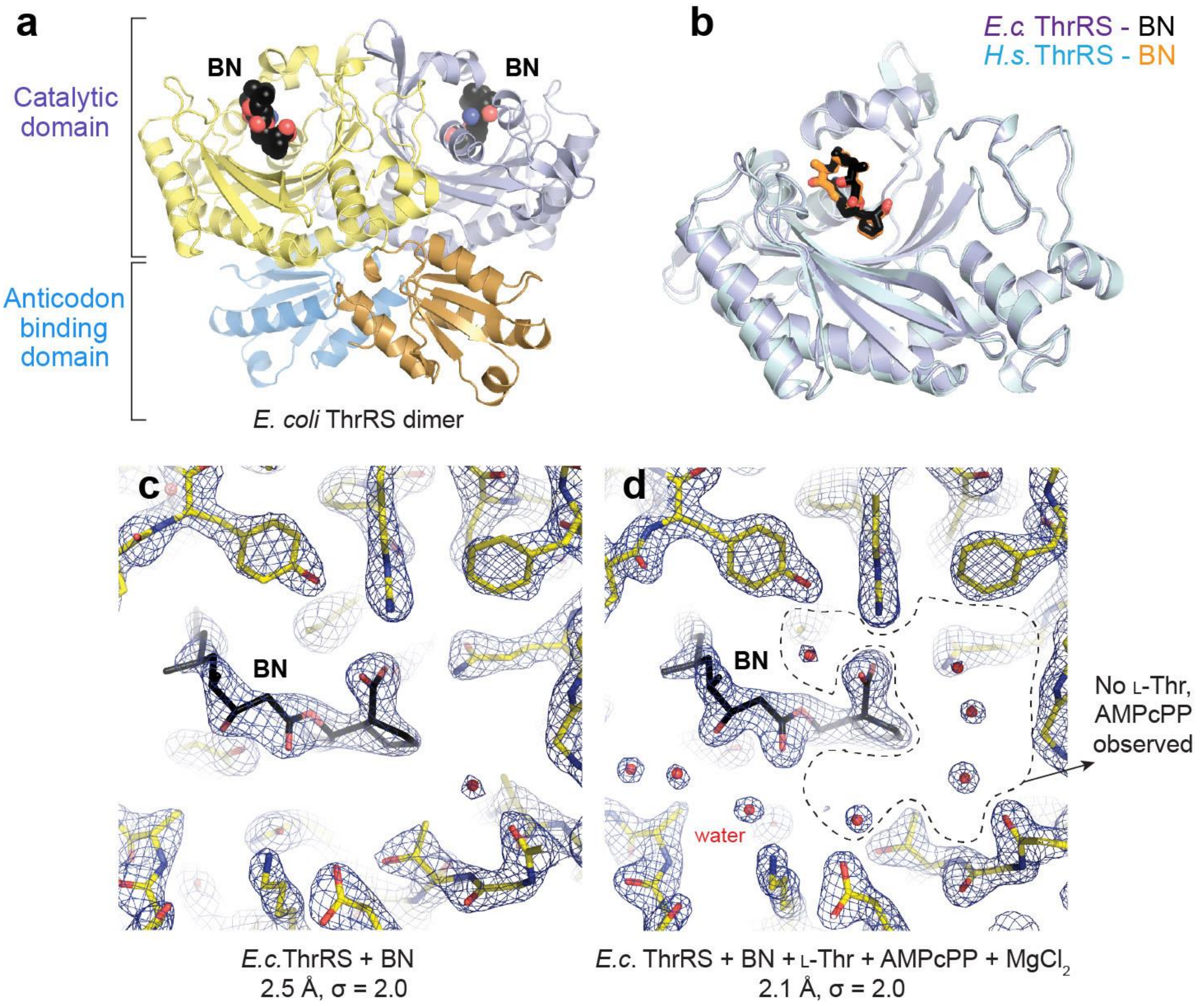

Supplementary Figure 3 | Complex structures of *E. coli* ThrRS–BN. (a) Overview of *E. coli* ThrRS-BN structure determined at 2.1 Å resolution. Protein is shown in cartoon and BN (black) is shown in sphere representation. **(b)** Structural superimposition of human and *E. coli* ThrRS-BN complex structures shows the close resemblance between the two BN-bound catalytic domains, with r.m.s.d. of 0.664 Å. **(c-d)** 2Fo-Fc electronic densities of *E. coli* ThrRS-BN structures determined from conditions with **(d)**, and without **(c)** 5 mM $_{L}$-Thr, 5 mM AMPcPP (ATP analog), and 10 mM $MgCl_2$, indicating BN does not co-bind with $_{L}$-Thr nor with ATP with ThrRS. In contrast, substrate mimicking Ser-AMS was co-crystallized with *E. coli* ThrRS with

the same crystal packing (PDB: 1FYF), supporting that BN may compete with L-Thr and AMPcPP in the crystal.

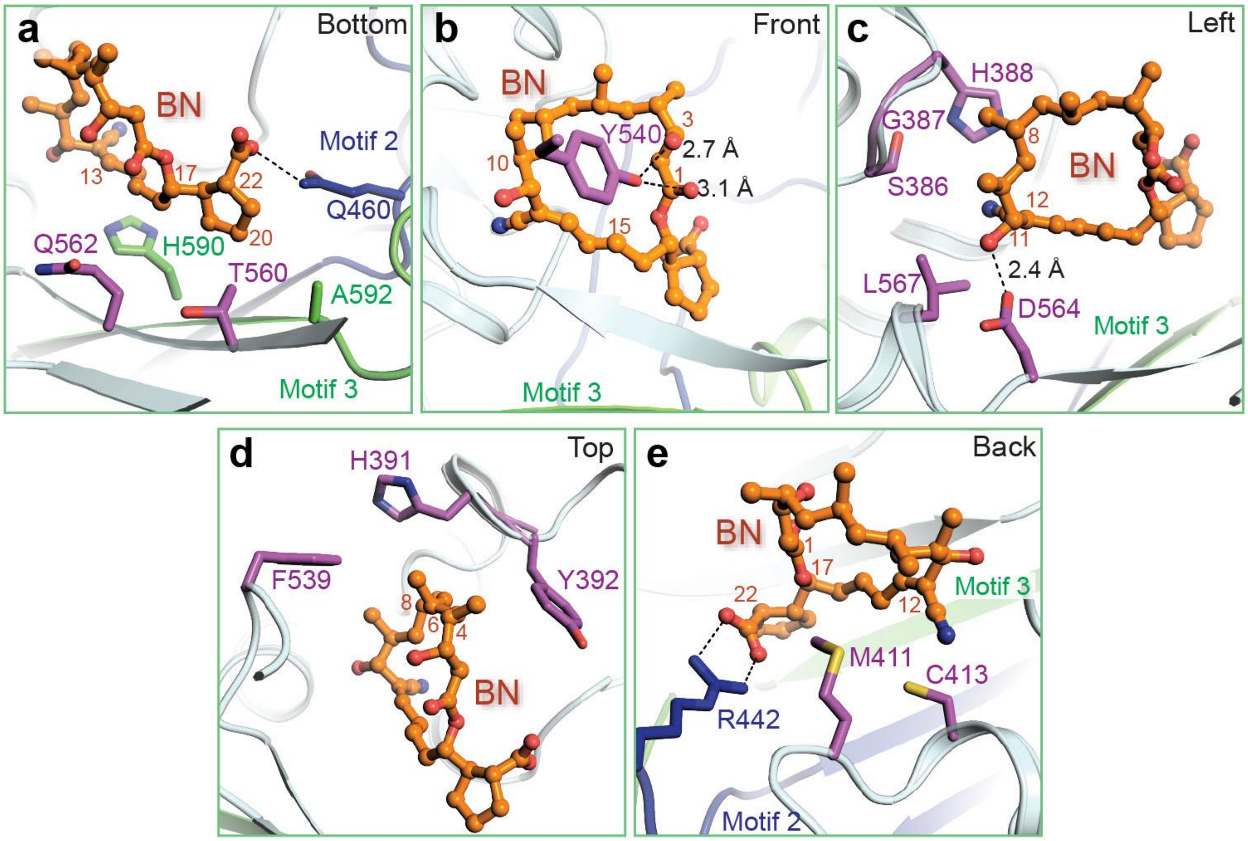

Supplementary Figure 4 | Detailed interactions between human ThrRS and BN. (a) At the bottom of BN, Q460 on motif 2 forms a H-bond with the carboxylic group of BN; H590, A592, T560 and Q562 from motif 3 and the antiparallel β strand of motif 3 form hydrophobic interactions with the C13-17 and C20 methylene groups of BN. **(b)** At the front, Y540 forms two H-bonds with hydroxyl groups at 1' and 3' and supports C10-15 portion of BN. **(c)** From the left, D564 interacts with 11' -OH group; L567, S386, G387, and H388 form Van der Waals contacts with the 8' methyl and 12' cyano groups of BN. **(d)** On the top, residues H391, Y392, and F539 contribute to the hydrophobic interactions with the C4-8 portion of BN. **(e)** At the backside, hydrophobic interactions are formed by M411 and C413. Side chain of M411 interacts with the 1'-17' ester group and the 22' carbonyl group. C413 interacts with the 12' cyano group. Particularly, the class II characteristic R442 forms bifurcated salt bridges with the 22' carboxylic group. These extensive interactions provide the high affinity of BN on ThrRS.

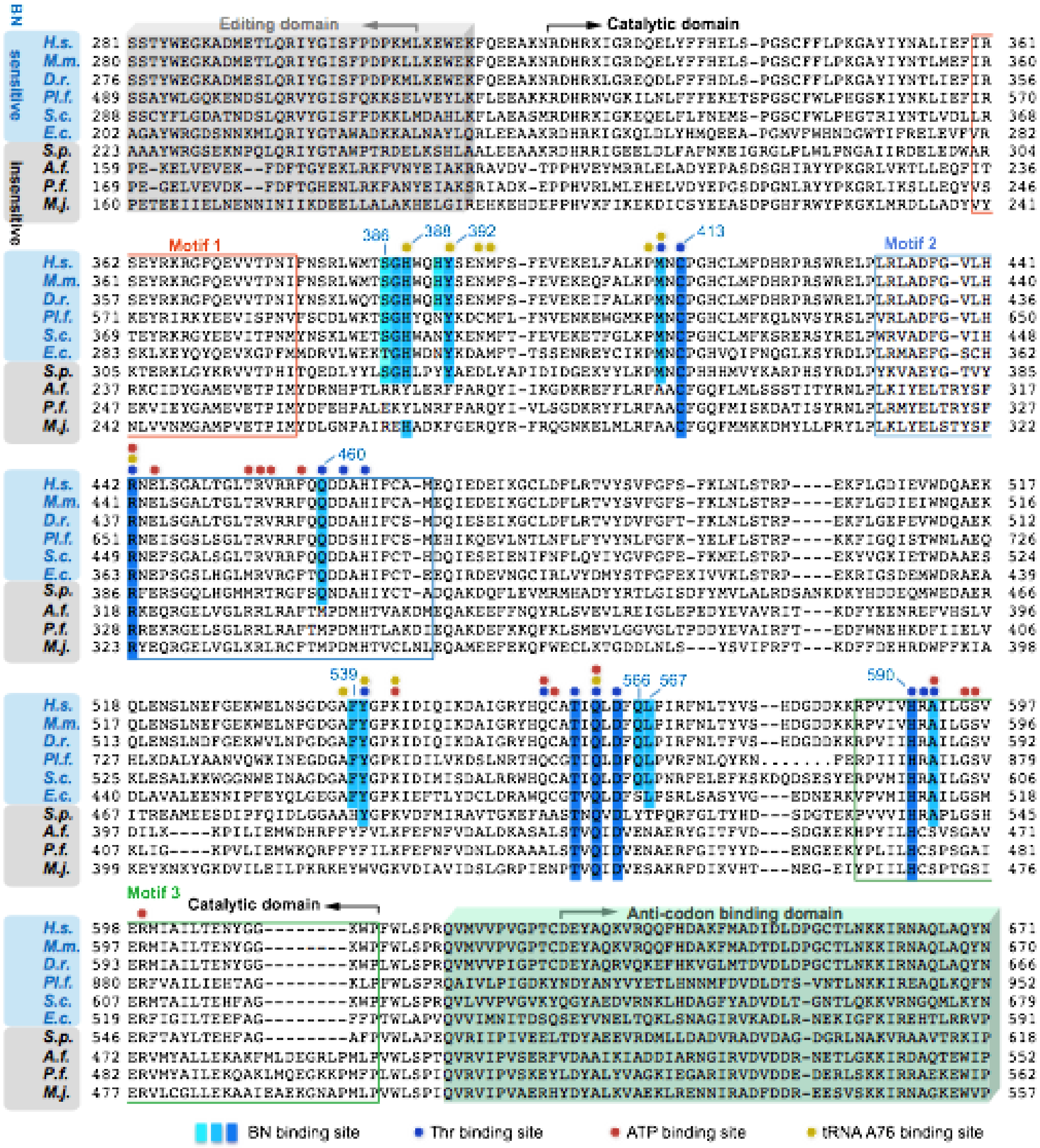

Supplementary Figure 5 | Sequence alignment of BN sensitive and insensitive ThrRSs. BN-interacting residues are highlighted in cyan to blue. Most of them are conserved in bacterial and eukaryotic ThrRSs, but not in archaeal ThrRSs and BN producing *Streptomyces parvulus*. *H.s., homo sapiens; M.m., Mus musculus; D.r., Danio rerio; Pl.f., Plasmodium falciparum 3D7; S.c.,*

Saccharomyces cerevisiae; E.c., Escherichia coli; S.p., Streptomyces parvulus; A.f., Archaeoglobus fulgidus; P.f., Pyrococcus furiosus; M.j., Methanococcus jannaschii. Only part of the anticodon binding domain sequences is shown for clarity.

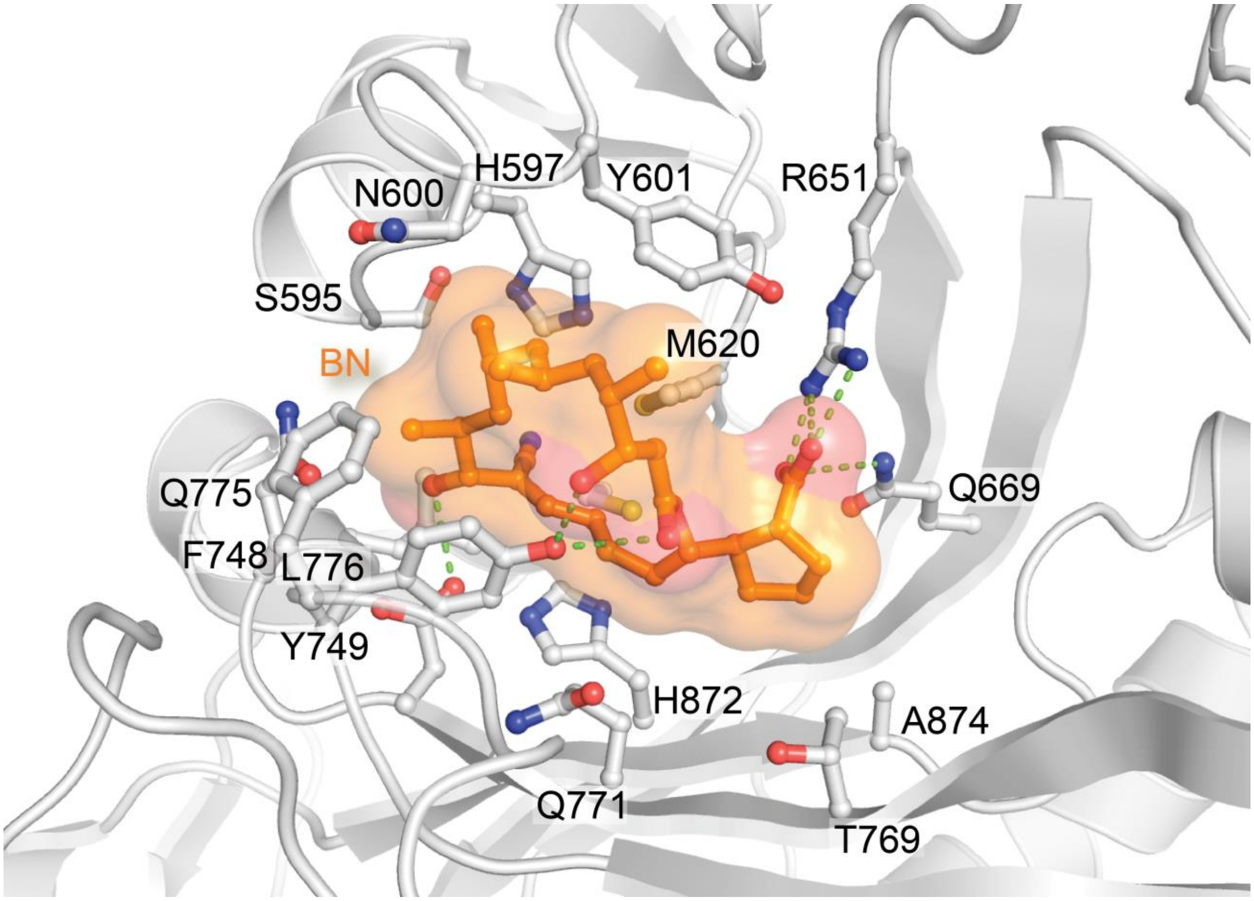

Supplementary Figure 6 | Structural model of *Plasmodium falciparum* ThrRS – BN complex. *Plasmodium falciparum* ThrRS structural model is colored in white. The BN (orange) interacting residues are shown as sticks.

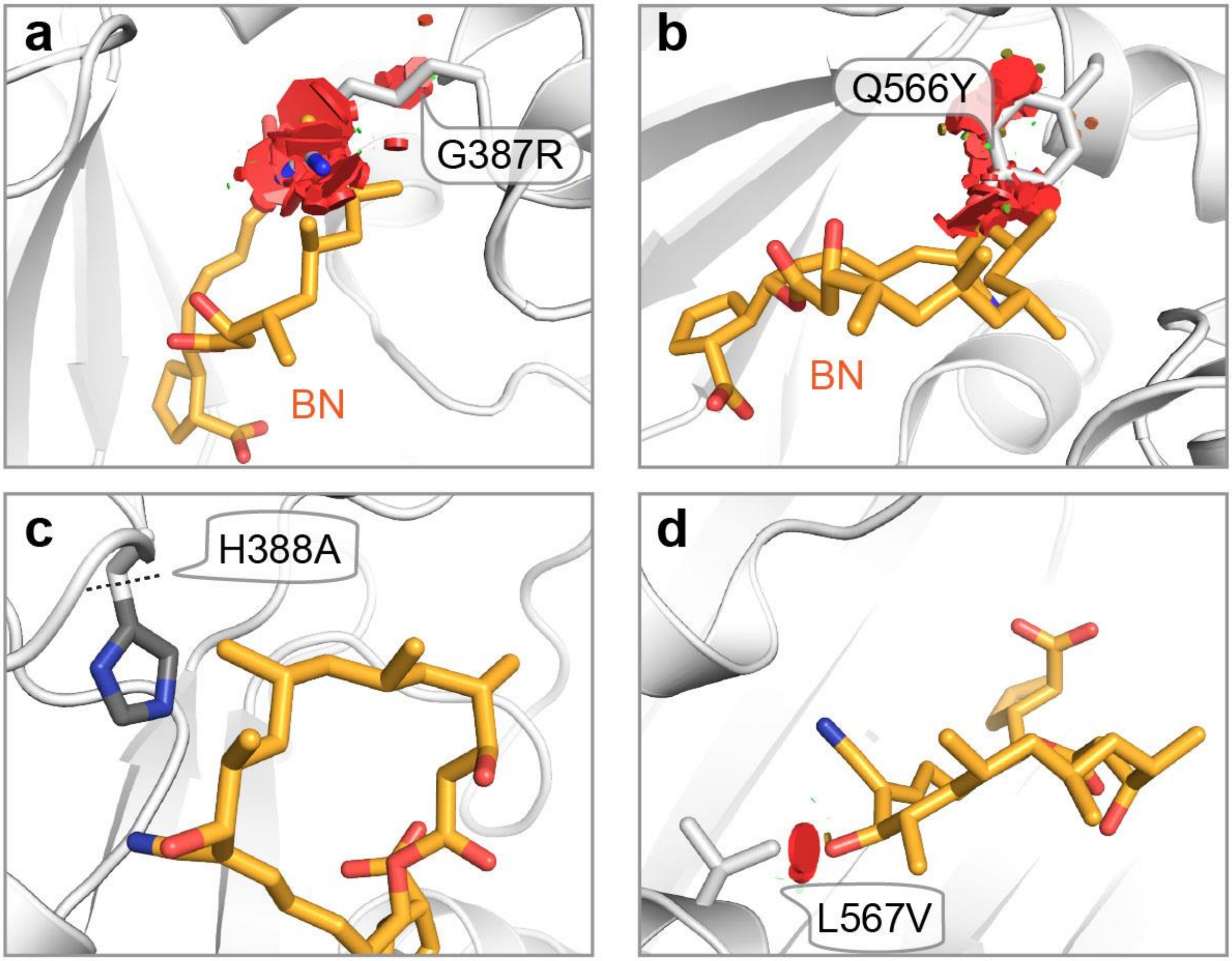

Supplementary Figure 7 | Structural mechanism of BN-resistance. **(a)** The G387R (numbering in human ThrRS) substitution in *A. fulgidus* ThrRS causes repulsion with the backbone atoms C7-8 of BN. Other commonly seen amino acid substitutions in archaeal ThrRS, such as S386R, S386E, G387K, and G387E form similar clashes with BN. **(b)** BN clashes with Q566Y substitution in self-resistant *Streptomyces parvulus* ThrRS (*Sp*ThrRS, or BorO). Other substitutions of the BN-binding residues in *Sp*ThrRS, such as H391Y, F539H, and L567T, can also contribute to the BN-resistance (Fig. 1d). **(c)** H388 forms van der Waals contact with the C7-methyl group of BN. The corresponding H309A ThrRS mutant showed 300-fold increased resistant to BN than wide type ThrRS in *E. coli*[1]. **(d)** L567 forms a close contact with the C10 hydroxyl group of BN. Mutation of L567V leads to BN-resistance in human ThrRS[2]. A

corresponding L489W ThrRS mutant in *E. coli* also caused a 1000-fold increased K_i than that of wide type ThrRS[1].

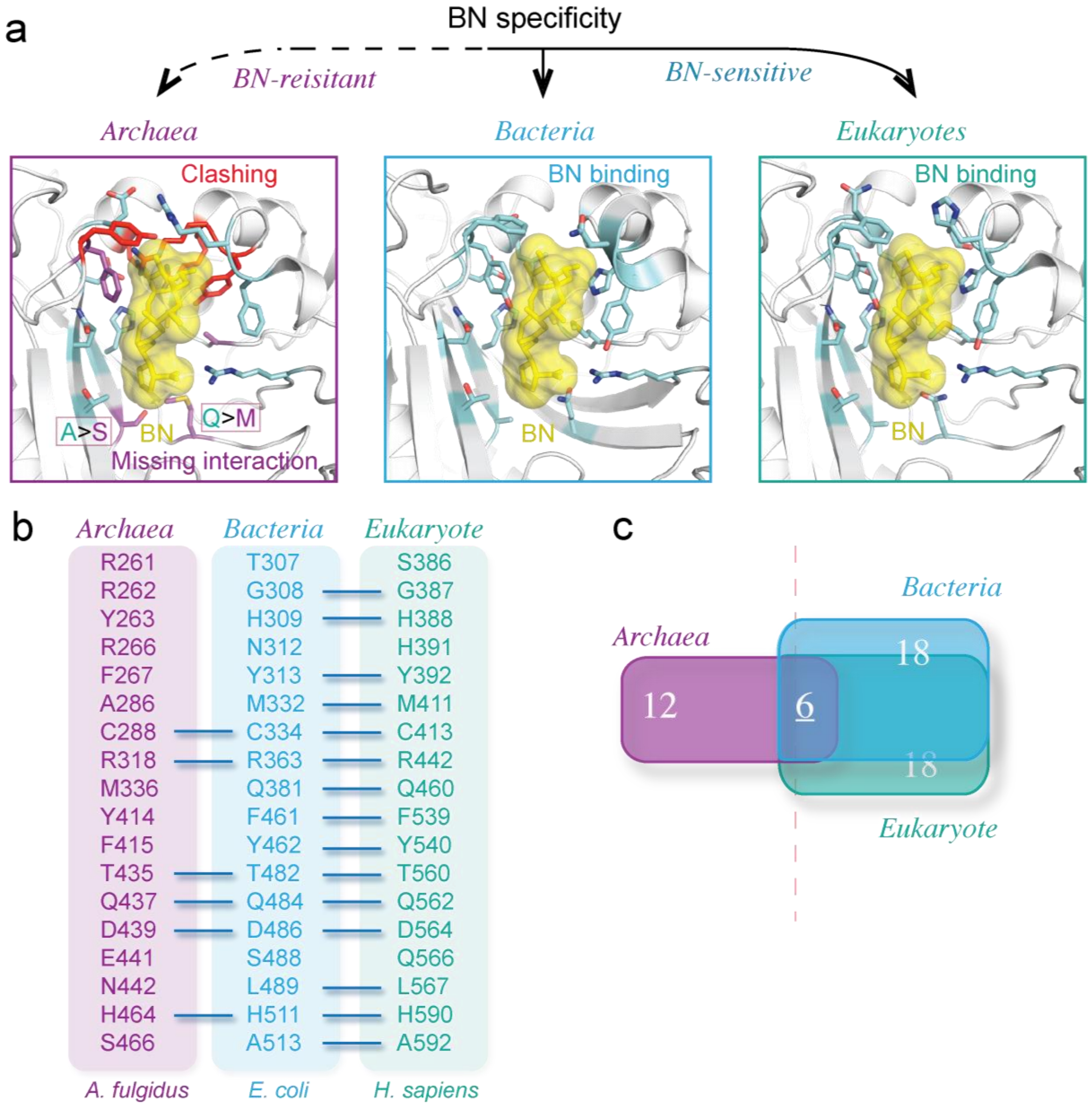

Supplementary Figure 8 | Kingdom specificity of BN on ThrRS's is mainly contributed by the fourth BN-binding site. **(a)** BN interacts with ThrRS through 18 residues. 15 of them are identically conserved between bacterial and eukaryotic ThrRS. In contrast, two thirds of these residues (12) are altered in archaeal ThrRS. Most of these altered residues (10 out of 12) are located at the fourth site for the macrolide structure of BN. These alterations cause loss of interaction and steric clash with BN, contributing to the resistance of archaea to BN. **(b)** The 18 *H. sapiens* and *E. coli* BN interacting residues, and the corresponding archaea *A. fulgidus* ThrRS

residues are listed. The identical residues across the species are linked by blue lines. **(c)** Diagram of the conservation of the 18 BN-binding residues in archaea, bacteria and eukaryotic ThrRS.

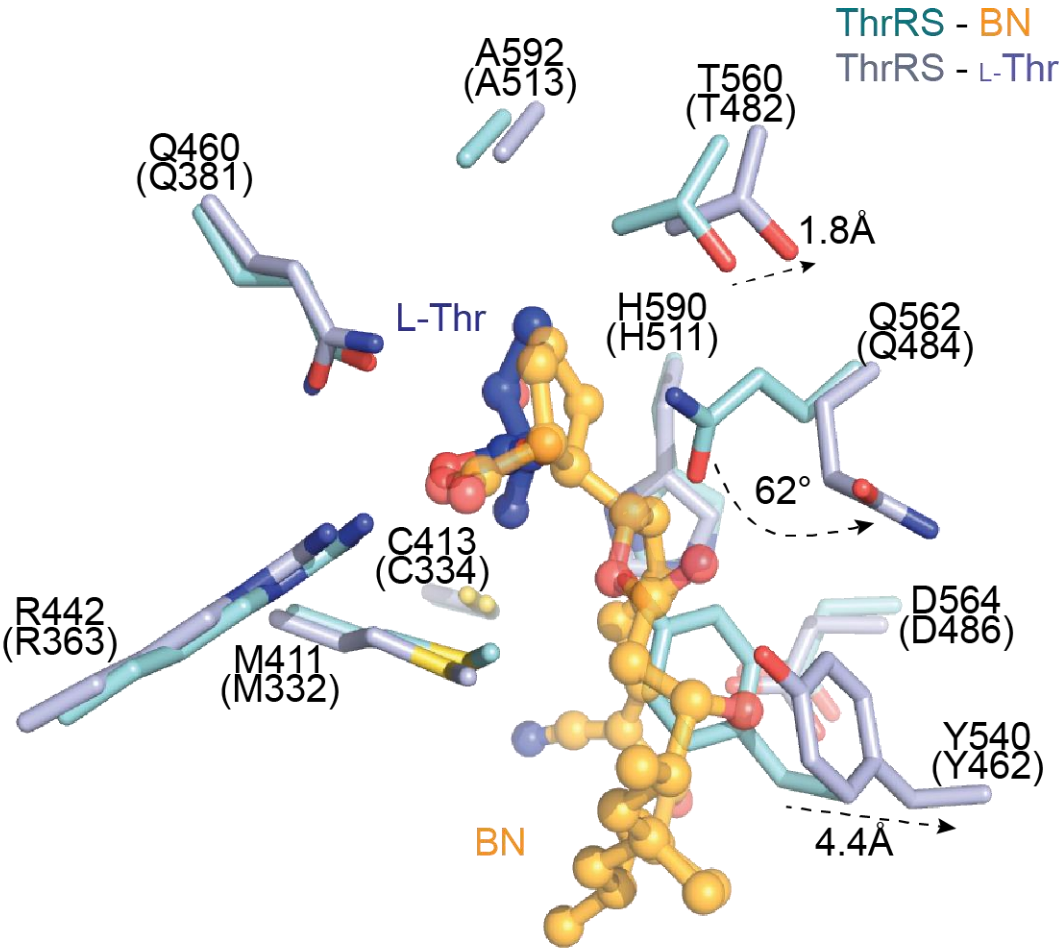

Supplementary Figure 9 | Residues involved in binding of both L-Thr and BN. 10 BN-interacting residues are involved in the interactions with L-threonine (L-Thr). In addition to steric clash, BN also triggers conformational changes at the L-Thr-binding site. T482 and Y462 shifted away from the ligand cleft by distances of 1.8 Å and 4.4 Å upon the binding of BN, respectively. Side chain of Q484 rotated 62° from pointing to the L-Thr-pocket to pointing to the edge of the pocket in order to accommodate BN. Both steric clashes and the observed conformational changes would cause BN to abolish the binding L-Thr by ThrRS.

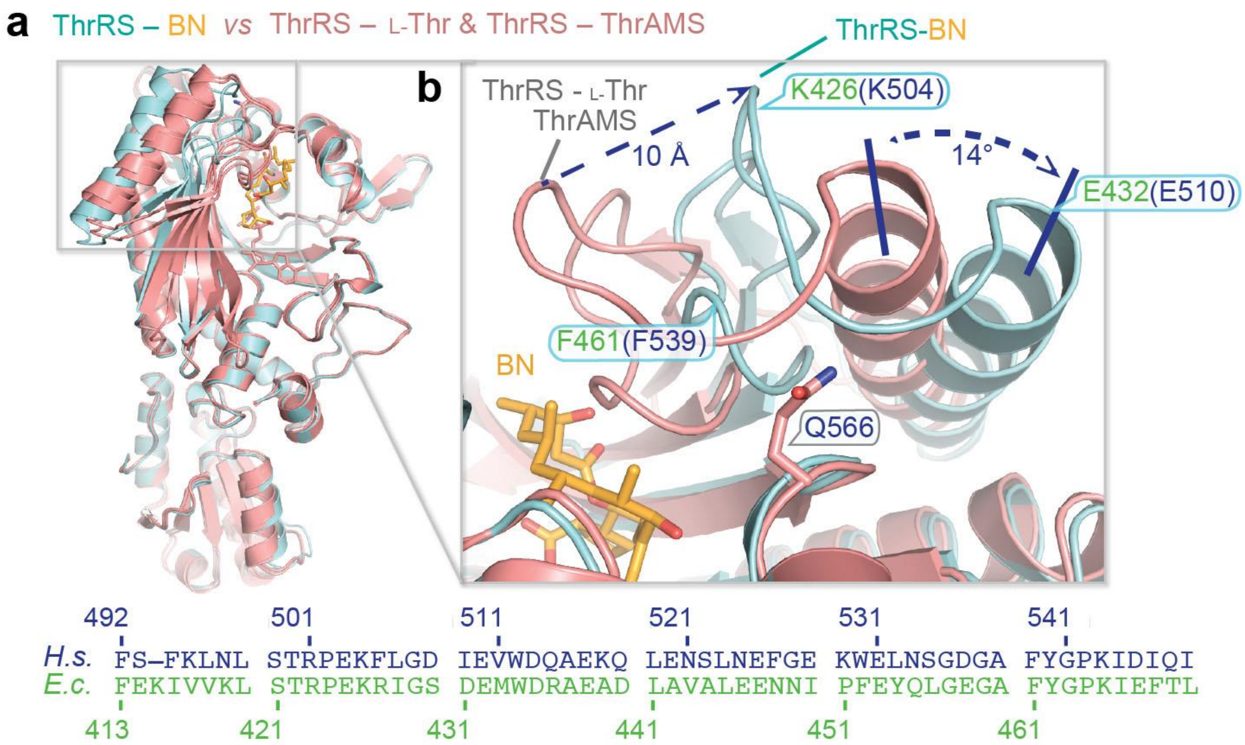

Supplementary Figure 10 | ThrRS–BN shows different structural changes from those with canonical substrates. (a) Large and distinct conformational changes of ThrRS are observed upon BN-binding (cyan. ThrRS-BN complex); in contrast, only small conformational changes of ThrRS are triggered by L-Thr, Thr-AMS (pink). **(b)** Zoom-in view of the BN-induced larger and wider conformational changes on residue 415-469. This region rotated by 14° with a largest shift of 10 Å. Corresponding sequences of the opening parts in human and *E. coli* ThrRS are aligned and colored in blue and green, respectively.

References:

1. Ruan, B. et al. A unique hydrophobic cluster near the active site contributes to differences in borrelidin inhibition among threonyl-tRNA synthetases. *J Biol Chem* **280**, 571-7 (2005).

2. Williams, T.F., Mirando, A.C., Wilkinson, B., Francklyn, C.S. & Lounsbury, K.M. Secreted Threonyl-tRNA synthetase stimulates endothelial cell migration and angiogenesis. *Sci Rep* **3**, 1317 (2013).
